# Impact of dysregulated microbiota-derived C18 polyunsaturated fatty acid metabolites on arthritis severity in mice with collagen-induced arthritis

**DOI:** 10.3389/fimmu.2024.1444892

**Published:** 2025-01-09

**Authors:** Katsuhiko Yoneda, Sho Sendo, Takaichi Okano, Hidenori Shimizu, Hirotaka Yamada, Keisuke Nishimura, Yo Ueda, Jun Saegusa

**Affiliations:** ^1^ Department of Rheumatology and Clinical Immunology, Kobe University Graduate School of Medicine, Kobe, Japan; ^2^ Noster Inc., Kyoto, Japan

**Keywords:** metabolome, rheumatoid arthritis, 16S rRNA, collagen-induced arthritis, polyunsaturated fatty acids, gut microbiota-derived metabolites

## Abstract

**Objective:**

We aimed to evaluate microbiome and microbiota-derived C18 dietary polyunsaturated fatty acids (PUFAs), such as conjugated linoleic acid (CLA), and to investigate their differences that correlate with arthritis severity in collagen-induced arthritis (CIA) mice.

**Methods:**

On day 84 after induction, during the chronic phase of arthritis, cecal samples were analyzed using 16S rRNA sequencing, and plasma and cecal digesta were evaluated using liquid chromatography–tandem mass spectrometry. Differences in microbial composition between 10 control (Ctrl) and 29 CIA mice or between the mild and severe subgroups based on arthritis scores were identified. The cecal metabolite profile and its correlation with the microbiome were evaluated with respect to arthritis severity.

**Results:**

The hydroxy and oxo metabolite levels were higher in CIA mice than in Ctrl mice, some of which, including 10-hydroxy-cis-6-18:1, were positively correlated with arthritis scores. The 9-trans,11-trans CLA levels in CIA mice had a negative linear correlation with arthritis scores. Microbial diversity was lower in severe CIA mice than in mild CIA or Ctrl mice. The abundance of *Lactobacillus* relatively increased in the severe subgroup of CIA mice compared with that in the mild subgroup and was positively correlated with arthritis severity.

**Conclusion:**

Alterations in gut microbiota and microbiota-derived C18 PUFA metabolites are associated in CIA mice and correlated with arthritis scores, indicating that plasma or fecal C18 PUFA metabolites can be potential biomarkers for arthritis severity and dysbiosis.

## Graphical Abstract

Alteration in microbiota-derived C18 PUFA metabolites and gut microbiota in CIA mice. The increased relative abundance of *Lactobacillus* correlated with arthritis severity. CLAs showed negative correlations with arthritis scores. This graphical abstract was created with BioRender. Yoneda, K. (2024) https://BioRender.com/m11c968.

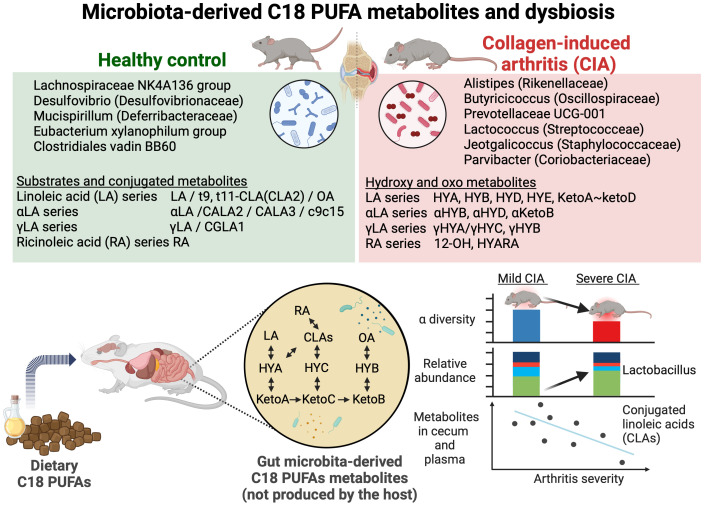

## 1 Introduction

The gut microbiota and microbiota-derived metabolites maintain gut homeostasis and regulate host metabolism, and their alterations are related to autoimmunity (1, 2). Gut dysbiosis and microbiota-derived metabolites can influence the pathophysiology of rheumatoid arthritis (3, 4).

Previous studies have described the changes in dietary lipid profiles containing some essential long-chain fatty acids, such as linoleic acid (LA), conjugated 9-trans,11-trans conjugated LA (CLA2), gamma LA (γLA), and oleic acid (OA) (5–9). Additionally, these supplements may prevent arthritis through anti-inflammatory effects (10–13).

Some C18 polyunsaturated fatty acids (PUFA), including LA and alpha-linolenic acid (αLA), are derived from diet. Specific gut microbial enzymes can bio-transform before metabolizing by the host from these dietary PUFAs into gut-microbiota-derived hydrophilic and oxidative metabolites through oxidation, hydroxylation, and saturation by specific anaerobic bacteria (14). Microbial modification of dietary PUFAs into bioactive metabolites could influence human health—for instance, species such as *Bifidobacterium* and *Lactobacillus* are known to produce conjugated linoleic acid hydrolysis enzymes (CLA-HY), which catalyze the conversion of LA into CLA, and activate G-protein coupled receptors, including GPR40 and GPR120, that trigger anti-inflammatory signaling pathways. CLA-producing enzymes are prevalent in the human microbiome, and the modulation of dietary PUFAs by gut microbiome enzymes may influence the susceptibility to inflammatory diseases, metabolic syndrome, and cardiovascular disease (15–18). The function of gut microbiome enzymes in response to dietary lipids is a key factor in explaining metabolic and autoimmune diseases (19). However, it remains an understudied area, and whether these microbiota-derived C18 PUFA metabolites are associated with rheumatoid arthritis and its disease activity remains unclear.

Hence, this study aimed to evaluate the gut microbiome and microbiota-derived C18 PUFAs, including their conjugated, hydroxy, or oxo fatty acids, and to investigate their correlation with arthritis severity in mice with collagen-induced arthritis (CIA) as a model of rheumatoid arthritis. To our knowledge, this is the first study to comprehensively analyze gut dysbiosis and microbiota-derived C18 PUFA metabolites and to integrate these findings not only with the presence of arthritis but also with its severity.

## 2 Materials and methods

### 2.1 Animals and diets

All male DBA/1JNCrlj mice (age, 6 to 7 weeks) were obtained from Jackson Laboratory Japan, Inc., and were randomly divided into two groups: the control (Ctrl) (*n* = 10) and CIA (*n* = 30) groups. Every five mice were housed in separate cages bedded with wooden tips. All mice had free access to solid, non-purified chows (DC-8, primary sources of lipids from soybeans, 23.1% crude protein, and 3,590 kcal per kilograms; CLEA Japan, Inc., Tokyo, Japan) and tap water. The mice were housed under specific pathogen-free conditions with a 12-h light/dark cycle and acclimatized to our facility for 7 days before immunization.

### 2.2 Induction of arthritis

Immunization Grade Bovine Type II Collagen (CII) at 2 mg per milliliter of 0.05 M acetic acid solution, complete Freund’s adjuvant (CFA), extinct *Mycobacterium tuberculosis* (H-37RA, 1 mg per milliliter), and lipopolysaccharide (LPS) from *Escherichia coli* 0111:B4 at 0.5 mg per milliliter (9028) were purchased from Chondrex Inc. (Redmond, WA, USA).

The mice, aged 7 to 8 weeks, received near the basement of their tail an intradermal injection of a collagen-emulsified solution containing 1 mg of CII per milliliter and 0.5 mg of CFA at a concentration of 100 μL per body. In the Ctrl group, the mice were injected with a vehicle of 0.025 M acetic acid.

On day 28, after receiving the immunization, the mice were intraperitoneally injected with 50 μg of LPS to induce and synchronize arthritis. The Ctrl mice were injected with phosphate-buffered saline as a vehicle.

### 2.3 Arthritis incidence and severity

The degree of macroscopic arthritis was graded as follows: 0, no evidence of erythema or swelling (no signs of arthritis); 1, erythema and mild swelling confined to either wrist/ankle joint, metacarpals/tarsals, or digits; 2, erythema and mild swelling extending from the wrist/ankle to the carpals/tarsals or inflammation of all digits without paw swelling; 3, erythema and moderate swelling extending from the wrist/ankle to the metatarsal joints, with swelling in some but not all digits; and 4, erythema and severe swelling encompassing the entire paw and all digits. The scores of all four paws were added for a composite score, with a maximum score of 16 per mouse.

The incidence of arthritis was defined as the percentage of mice that developed an arthritis score of 1 or higher, as scored above.

Paw thickness was measured using a digital caliper on days 14, 28, 33, 42, 56, 70, and 84. The maximum width of the forepaws and the hind paws was measured at the wrist and ankle joints, respectively. The sum of these values of all four paws was used to assess changes in paw swelling over time. Paw thickness is expressed as the change in average of four-paw thickness relative to day 14.

Investigators could not be blinded to the CIA in the mice owing to the arthritis, but the arthritis score and paw measurements were performed by two independent experimenters.

### 2.4 Plasma sampling

On day 84, blood was collected under anesthesia from the retro-orbital sinus using microcapillary tubes. The samples were inserted into Microtainer^®^ PST™ Lithium Heparin blood collection tubes (365985, Becton, Dickinson and Company, Franklin Lakes, NJ, USA) and quickly centrifuged at 12,000 × *g* for 2 min at 4°C. All plasma samples were stored at –80°C for further analysis.

### 2.5 Metabolomic analyses

#### 2.5.1 Lipid extraction from plasma and cecal samples

On day 84, plasma and cecal specimens were collected from CIA (*n* = 29) and Ctrl (*n* = 10) mice.

The frozen cecal samples were added to homogenization tubes with metal corn kept frozen in liquid nitrogen and homogenized using a multi-bead shocker (MB-3200C[S]; Yasui-kikai, Osaka, Japan). Next, the resulting homogenate was filled with methanol at 50 mg/mL (134-14523, FUJIFILM Wako Pure Chemical Corporation [Wako], Osaka, Japan) and stored at −30°C overnight. After centrifuging at 3,500 × *g* for 10 min at 4°C to eliminate the debris, these supernatants (100 μL) were mixed with 100 μL of deionized water added with 10 μL of internal standard (for reference) and were vortexed. After centrifuging at 13,000 × *g* for 5 min at 4°C, these supernatants were collected to pass through the conditioned column.

In total, 20 μL of plasma samples were mixed with methanol to 400 μL and stored at −30°C overnight. After centrifuging at 15,000 × *g* for 10 min at 4°C, 300 μL of the resulting supernatant was transferred to tubes containing 300 μL of deionized water and 7.5 μL of internal standard (for reference) and was vortexed.

MonoSpin C18-AX (GL Science, Tokyo, Japan) was used for solid-phase extraction. Subsequently, 300 μL of methanol was added to the column and centrifuged (3,500 × *g* for 1 min). This process was repeated with deionized water, and the prepared sample solution was added and centrifuged (3,500 × *g* for 2 min) and washed with 300 μL water and 50% methanol, and 100 μL of the sample was eluted with an elution buffer (acetic acid/methanol/water = 2:90:8).

#### 2.5.2 Liquid chromatography–tandem mass spectrometry analysis

The C18 PUFA metabolites were analyzed using a Nexera X3 HPLC system (Shimadzu, Kyoto, Japan) coupled with an LCMS-8060NX triple quadrupole mass spectrometer (Shimadzu). A detailed description of the metabolomic analysis method has been presented previously (20).

#### 2.5.3 Metabolomic data analysis

The principal component analysis (PCA) plot and heatmap of metabolites were generated using MetaboAnalyst version 5.0 (21), and a volcano plot was drawn using VolcaNoseR (22) with a fold change threshold of >1.0 or <−0.5 and an adjusted *p*-value (*q*-value) threshold of <0.01.

### 2.6 Microbiome analyses

#### 2.6.1 Gut microbial composition

The cecal specimens with intestinal contents were harvested, rapidly shock-frozen with liquid nitrogen, and stored at –80°C. The total microbial genomic DNA was extracted from these cecal contents using a QIAamp PowerFecal Pro DNA Kit (QIAGEN, Germany). The concentrations of the DNA samples were quantified using NanoDrop™ Lite (Thermo Fisher Scientific, Waltham, MA, USA). The V3–V4 region of bacterial 16S rRNA gene was amplified using polymerase chain reaction with KAPA HiFi HotStart ReadyMix (2×) (Takara Bio, Inc., Kapa Biosystems, Wilmington, MA, USA), which contains the following primers: forward, 341F (5′-TCGTCGGCAGCGTCAGATGTGTATAAGCGACAGCCTACGGGNGGCWGCAG-3′), and reverse, 805R (5′-GTCTCGTGGGCTCGGAGATGTGTATAAGAGACAGGACTACHVGGGTATCTAATCC-3′), with overhang for Illumina Miseq^®^. The amplified template DNA library was prepared using a Nextera XT index Kit v2Set A (Illumina, San Diego, CA, USA), and purified 16S rRNA gene sequencing was performed using MiSeq^®^ sequencer (Illumina, San Diego, CA, USA) and MiSeq Reagent kit v3 (600 cycles), per the manufacturer’s instructions.

#### 2.6.2 Bioinformatic analysis and taxonomic identification

Sequencing reads were analyzed using the Quantitative Insights Into Microbial Ecology 2 (https://qiime2.org/) software package (23). Operational taxonomic unit (OTU) classification was performed based on 97% sequence similarity, searching the representative sequence set against the database using the best hit. Briefly, reads were paired, and taxonomic information was mapped to an OTU using the SILVA database (version SSU138.1) (24). Data were visualized using R version 4.1.2.

Alpha diversity indices were used to evaluate within-sample richness and evenness at the feature level. Beta diversity dissimilarity comparisons (based on the Bray–Curtis index and Jensen–Shannon divergence) were tested through analysis of similarities and permutational multivariate analysis of variance with the plugin of MicrobiomeAnalyst 2.0 (25). Linear discriminant analysis effect size (LEfSe) was used to detect significant variations in the abundant taxa across the groups. Taxa were statistically significant (*p* < 0.05) and had a linear discriminant analysis (LDA) score of >2.0, which indicated an adequate effect size. SparCC network analysis was performed to decipher the association between the bacteria and the severity of arthritis (26), with a low variance filter by 30% of elimination based on the interquartile range to remove low-quality or uninformative features. Random forest analysis was also performed as a supervised model to reveal variations in the microbiota among distinct groups using MicrobiomeAnalyst 2.0 (25). Tax4fun2 was used to simulate functional gene redundancy in microbial communities (27).

### 2.7 Statistical analyses

All statistical tests were performed using bilateral tests, and data were analyzed using GraphPad Prism version 10.0.0 and R statistical environments. The nonparametric data are presented as medians ± interquartile ranges and compared using the Mann–Whitney *U*-test and Kruskal–Wallis test with multiple-comparison tests by controlling the false discovery rate using the Benjamini and Hochberg method. Repeated-measures data are shown as means ± standard deviations and were analyzed using two-way repeated-measures analysis of variance with Sidak’s multiple-comparison tests. *P*-values <0.05 were considered to indicate statistical significance.

## 3 Results

### 3.1 Clinical course of CIA mice

**Figure 1:**
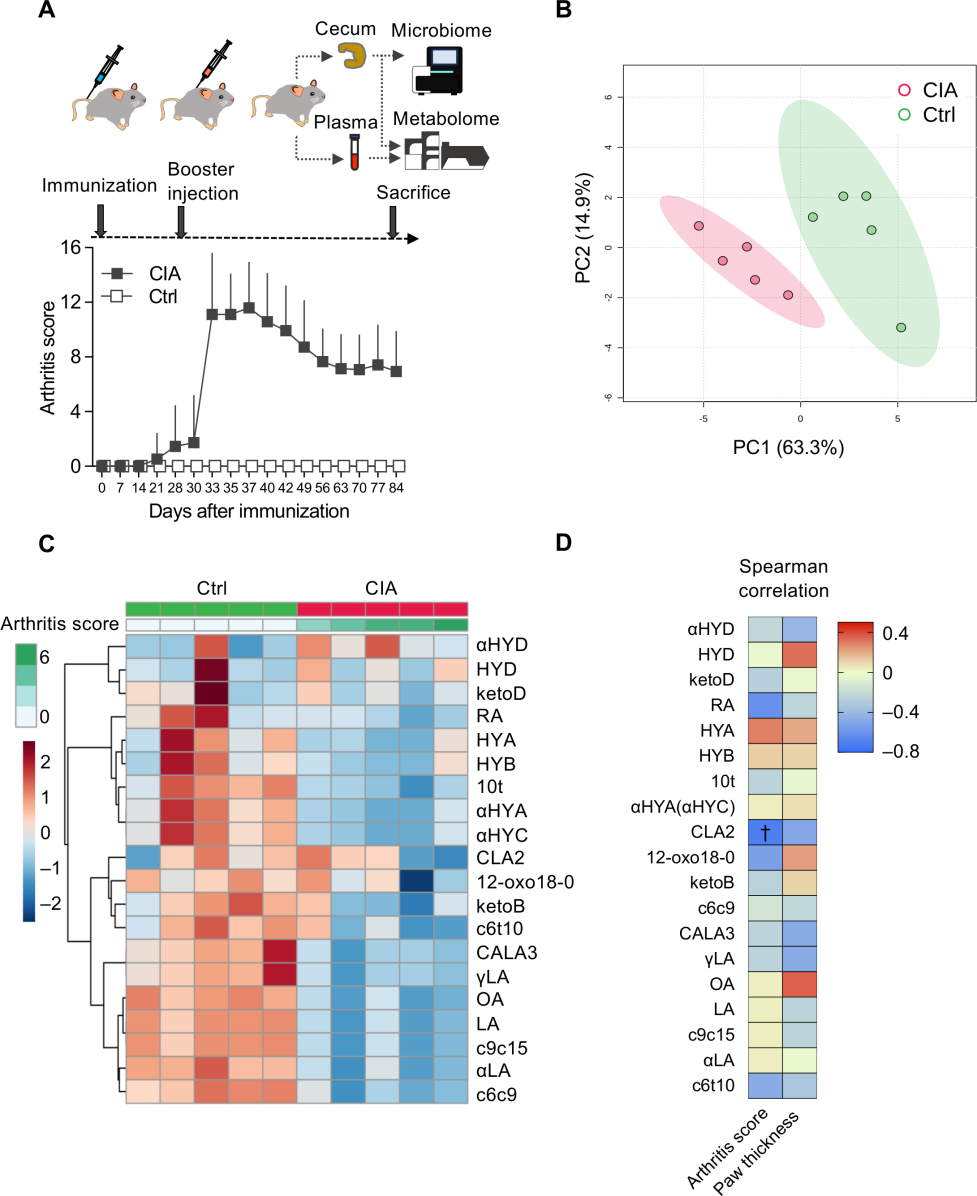
Plasma microbiota-derived C18 dietary PUFAs are altered in CIA mice. **(A)** The clinical course of CIA mice (gray boxes, *n* = 29) was compared with that of control (Ctrl) mice (white boxes, *n* = 10) and experimental overview. A total of 30 DBA/1J mice were immunized by intradermal inoculation with bovine type II collagen and complete Freund’s adjuvant on day 0. On day 28, the mice were intraperitoneally injected with lipopolysaccharide (LPS) to synchronize the onset of arthritis. Unfortunately, on day 30, one of the CIA mice died because of heightened susceptibility to LPS. Plasma and cecal samples were collected from 29 CIA and 10 Ctrl mice on day 84. Arthritis scores are described as means ± standard deviations. **(B)** Comparison of the plasma metabolome between the CIA (red dots, *n* = 5) and control mice (green dots, *n* = 5) using principal component analysis (PCA) score plots. **(C)** Heatmap illustrating changes in plasma metabolite abundance between the CIA (*n* = 5) and Ctrl (*n* = 5) mice. Hierarchical clustering (Euclidean distance, Ward linkage method) is shown. **(D)** Correlation between arthritis scores/paw thickness and plasma metabolite levels. The density of red or blue indicates which variables have high correlation coefficients with arthritis scores/paw thickness (positive or negative, respectively) (Spearman’s rank correlation coefficient). ^†^ *p*-value < 0.05.

An experimental overview is summarized in **Figure 1A**. DBA/1J mice showed spontaneous onset of arthritis on and after day 21 from immunization. After receiving a booster injection of LPS on day 28, their arthritis scores promptly aggravated and synchronized with the levels of arthritis that peaked on days 35–40. The CIA mice gradually improved on day 40 (**Figure 1A**; **Supplementary Figure S1**).

### 3.2 Plasma metabolomic analyses displayed the altered microbiota-derived C18 dietary PUFAs

Using the metabolomics method by liquid chromatography–tandem mass spectrometry, 20 metabolites in the plasma and 50 metabolites in the cecal digesta were quantified and are tabulated in **Supplementary Table S1**. We identified different characteristics in the plasma samples of the CIA (*n* = 5) and Ctrl (*n* = 5) mice.

PCA and a heatmap of plasma metabolites showed an apparent separation between CIA mice and controls (**Figures 1B, C**). The levels of almost all plasma metabolites and their hydroxy, oxo, and conjugated isomers decreased in the CIA group, except for 13-hydroxy-cis-9, cis15-18:2 (αHYD) (**Figure 1C**).

Spearman’s correlation analysis revealed a relationship between arthritis scores and plasma metabolites (**Figure 1D**; **Supplementary Table S2**). Plasma CLA2 was significantly inversely correlated with the arthritis scores.

### 3.3 Digested cecal metabolites from CIA mice showed different characteristics in microbiota-derived C18 PUFAs

**Figure 2:**
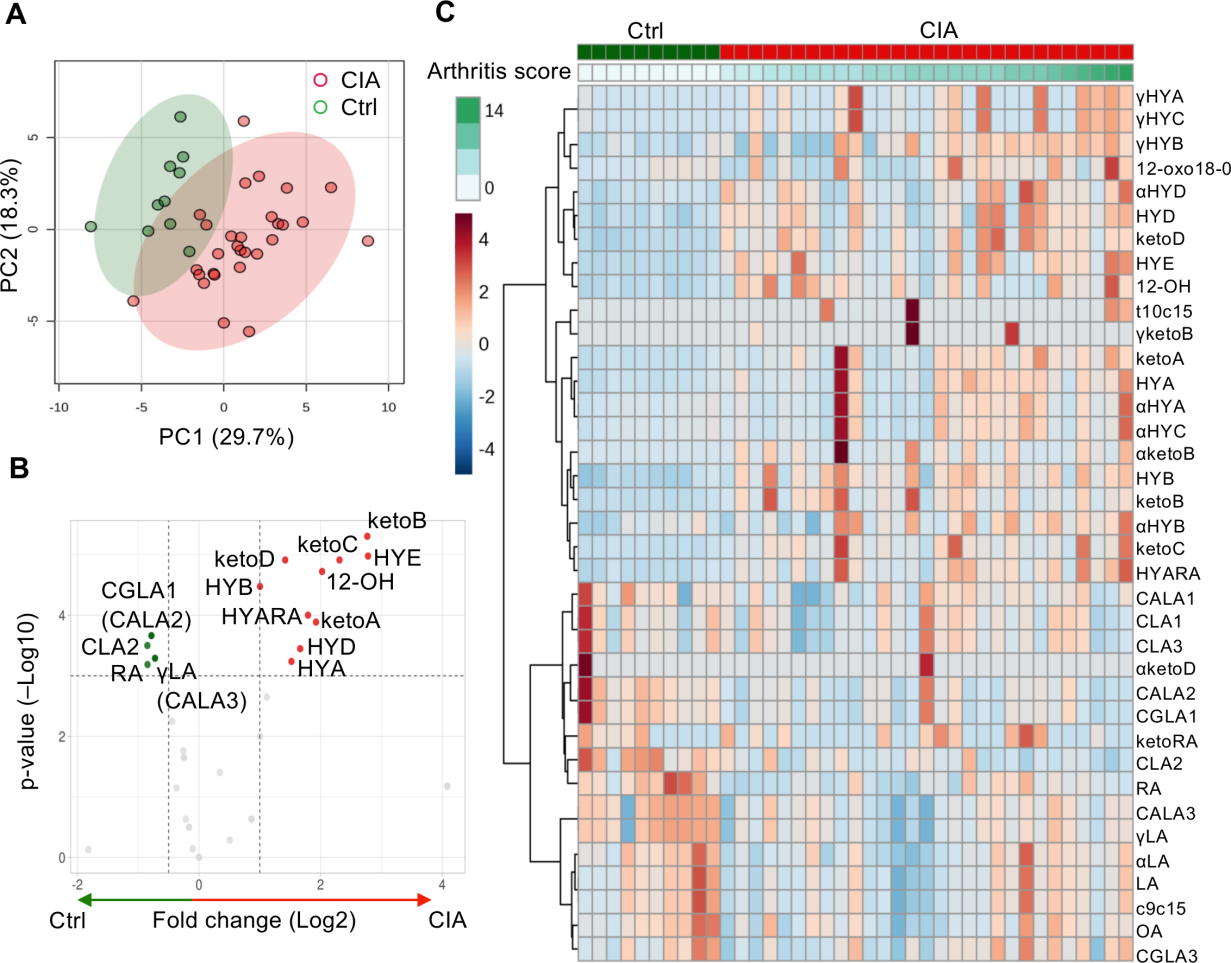
Significantly altered C18 PUFAs and their microbiota-derived metabolites in the cecum between the Ctrl and CIA groups. **(A)** Score plot of cecal metabolites with PCA (red for the CIA group, *n* = 29; green for the Ctrl group, *n* = 10). **(B)** Volcano plot of cecal metabolites. It can highlight some distinct components for hydroxy fatty acids (HYA, HYB, 12-OH, HYD, HYE, and HYARA) or oxo fatty acids (ketoA, ketoB, ketoC, and ketoD) in the CIA group (red) and conjugated fatty acids (CLA2 and CGLA1) or substrates (RA and γLA) in the Ctrl group (green). The fold-change threshold was 1.5, and the adjusted *p*-value (*q*-value) threshold was 0.01. **(C)** Heatmap analysis of cecal metabolites. The row displays the metabolites, and the column represents each sample. The color scale corresponds to the magnitude of the difference compared with the average value. Please refer to the manuscript for each abbreviation of isoforms and metabolites. The figures in **(A, C)** were generated using MetaboAnalyst version 5.0.

As we found a difference in plasma metabolites between the two groups, we also explored the differences in cecal metabolites between CIA (*n* = 29) and Ctrl (*n* = 10) mice. The PCA of cecal metabolites demonstrated different variations between the two groups (**Figure 2A**).

**Figures 2B, C** show the differentially abundant cecal metabolites identified in the CIA mice compared with the Ctrl mice. The fold change thresholds of  >1.0 or <−0.5 and adjusted *p*-value (*q*-value) of <0.05 were defined to indicate significantly different metabolites in **Figure 2B** (**Supplementary Table S3**). The levels of microbiota-derived components of hydroxy fatty acids (HYA), 10-hydroxy-18:0 (HYB), 13-hydroxy-cis-9-18:1 (HYD), 10,13-dihydroxy-18:0 (HYE), and 12-hydroxy-18:0 (12-OH) or oxo fatty acids (10-oxo-cis12-18:1 [ketoA], 10-oxo-18:0 [ketoB], 10-oxo-trans11-18:1 [ketoC], and 13-oxo-cis9-18:1 [ketoD]) were high in the CIA group. The metabolites with the most significantly higher levels in the CIA cecum were hydrated to 10- or 13-hydroxyl C18 PUFAs. However, the cecal concentrations of C18 PUFA substrates, ricinoleic acid (RA) and γLA or their conjugated fatty acids, cis-6, cis-9, trans-12-18:3/trans-9, trans-11, cis-15-18:3, CLA2, and trans-10, cis-12, cis-15-18:3, were relatively higher in Ctrl mice than in CIA mice.

Microbes converted PUFAs into several PUFA metabolites, such as LA- (**Supplementary Figure S2A**), αLA- (**Supplementary Figure S2B**), γLA- (**Supplementary Figure S2C**), and RA-derived metabolites (**Supplementary Figure S2D**). These metabolite maps illustrated the direction of transformation of these unique PUFAs catalyzed by anaerobic bacteria, and the differences in cecal metabolites between the two groups indicated that CIA mice were rich in hydroxylated and oxo fatty acid metabolites. Some cecal metabolites, such as LA, CLA2, γLA, and OA, showed similar patterns as those in the plasma analysis (**Supplementary Table S4**).

**Figure 3:**
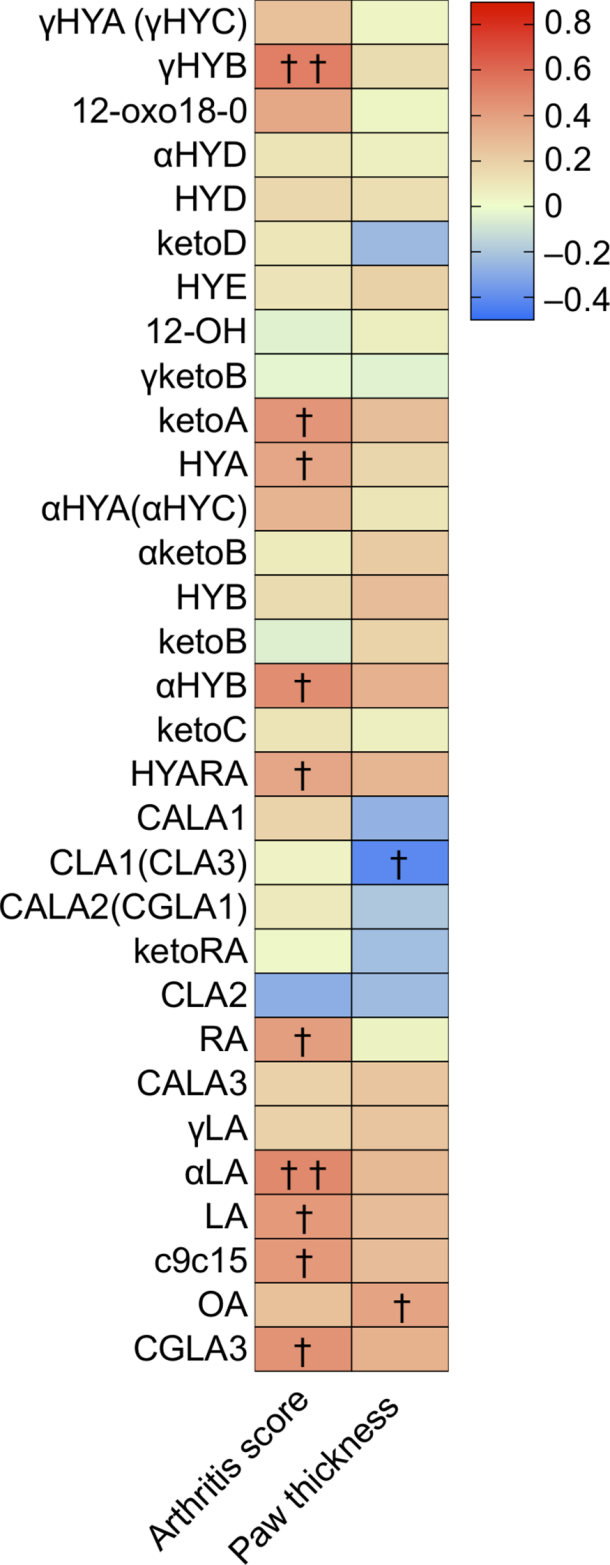
Cecal metabolites were correlated with arthritis score and paw thickness, especially several hydroxy and oxo fatty acids. Heatmap displaying the correlation between arthritis scores/paw thickness and cecal metabolite levels. The density of the red or blue bar chart shows which variables have high correlation coefficients with arthritis scores (positive or negative) (Spearman rank correlation coefficient). ^†^ *p*-value < 0.05, ^††^ *p*-value < 0.01.

The left side of **Figure 3** (**Supplementary Table S5**) shows significant correlations between arthritis scores and hydroxy or oxo C18 PUFAs containing at least a cis bond at the 6, 9, 12, and 15 positions, excluding 12-oxo18-0. Particularly, 10-hydroxy-cis6-18:1 (γHYB) in γLA intermediates, αHYB in αLA intermediates, HYA and ketoA in LA intermediates, and HYARA in RA intermediates, 10-hydroxy, or oxo C18 PUFAs could distinguish arthritis severity with a positive correlation. These values increased in the CIA group (**Supplementary Figures S2A–C**). Furthermore, the levels of LA, αLA, and RA (C18 PUFA substrates) had a significant positive correlation with arthritis scores, whereas the levels of these substrates were lower in CIA mice than in Ctrl mice (**Supplementary Figures S2A–C**). An unusual fatty acid, cis-9, cis-15-octadecadienoic acid, and cis-6, trans-10, cis-12-octadecatrienoic acid also had a significant positive correlation with arthritis scores.

Moreover, the right side of **Figure 3** (**Supplementary Table S5**) shows significant correlations between the four paw thicknesses and hydroxy or oxo C18 PUFA metabolites. The 9-cis,11-trans and 10-trans,12-cis CLA (CLA1 and CLA3, respectively) were significantly and inversely correlated with paw thickness. Similar to the levels of C18 PUFA substrates, OA showed a significant positive correlation with paw thickness.

Although there was no significant correlation between CLA2 and arthritis scores or paw thickness according to Spearman’s correlation analysis, the regression lines of plasma and cecal CLA2 levels tended to indicate a negative linear correlation with arthritis severity (**Supplementary Figure S3**).

### 3.4 Differential abundance of cecal microbiota in CIA mice determined using 16S rRNA sequencing

**Figure 4:**
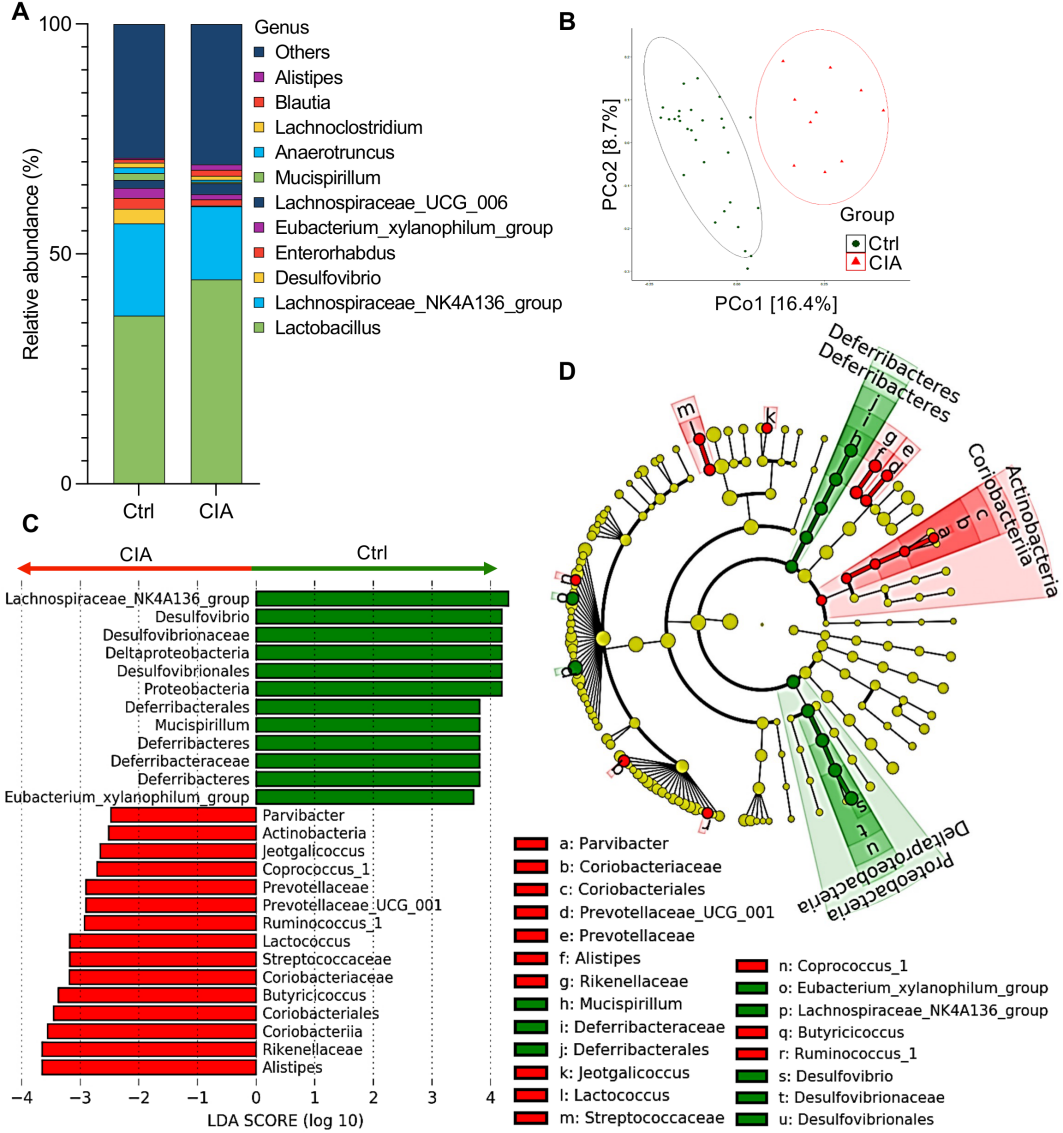
Cecal microbiota analysis in CIA mice shows species abundance and differentials. **(A)** The taxonomic bar plot shows the altered composition of the cecal microbiota in CIA mice compared with that in Ctrl mice. The relative abundance at the genus level is shown as the mean (Ctrl group, *n* = 10; CIA group, *n* = 29). **(B)** Principal coordinate analysis (PCoA) plot illustrating beta-diversity-based weighted UniFrac distance comparison of cecal microbiota from CIA and Ctrl mice. Ellipses indicate 95% confidence intervals. **(C)** Linear discriminant analysis (LDA) scores from linear discriminant analysis effect size (LEfSe) analyses were assessed on relative OTU abundances, depicting the most differentially abundant taxa between the cecal microbiomes of CIA (red) and Ctrl (green) mice. **(D)** The cladogram plotted from the LEfSe analysis indicates differences in the relative abundance of cecal microbes. Green indicates taxa enriched in the Ctrl mice, whereas red indicates taxa enriched in the CIA mice. From the inside to the outside, the circles represent five levels: phylum, class, order, family, and genus. Data in **(C, D)** show taxa with LDA scores >2 and *p* < 0.05 in the Wilcoxon signed-rank test.

We analyzed the cecal contents using 16S rRNA sequencing to distinguish the intestinal microbiota between CIA and Ctrl mice. Taxonomic differences at the genus level showed altered microbiota composition in CIA mice compared with that in Ctrl mice (**Figure 4A**).

To estimate the differences in the microbiome composition between the two groups, we performed principal coordinate analysis using weighted UniFrac, which revealed that the Ctrl and CIA mice possessed different communities in the cecum (**Figure 4B**).

To identify specific taxa that were differentially abundant between the two groups, we performed linear discriminant analysis (LDA) effect size (LEfSe), which highlighted several genera with a significantly higher relative abundance in CIA mice compared to Ctrl mice. Specifically, the following genera/families were enriched in CIA mice: *Parvibacter* (family Coriobacteriaceae), *Alistipes*, *Prevotellaceae UCG-001*, *Lactococcus*, *Jeotgalicoccus*, *Coprococcus 1*, *Butyricicoccus*, and *Ruminococcus 1* (**Figures 4C, D**). In contrast, the Ctrl group showed an increased abundance of Lachnospiraceae NK4A136, *Desulfovibrio*, *Mucispirillum*, and *Eubacterium xylanophilum*.

Next, to identify alterations in taxa that were differentially abundant across the two groups of samples involving individual variations, we performed LEfSe analysis to evaluate the LDA score and cladogram (**Figures 4C, D**, respectively). Although *Lactobacillus* was the most abundant taxon in the taxonomic bar plot of **Figure 4A**, the LDA scores indicated that the following eight genera or families showed the highest relative abundance in CIA mice: (1) *Parvibacter*, which originates from the family *Coriobacteriaceae* in the phylum of Actinobacteria, (2) *Alistipes*, (3) *Prevotellaceae UCG-001*, (4) *Lactococcus*, (5) *Jeotgalicoccus*, (6) *Coprococcus_1*, (7) *Butyricicoccus*, and (8) *Ruminococcus_1*.

In contrast, four genera or families showed the highest relative abundance in the Ctrl group: (1) *Lachnospiraceae NK4A136*, (2) *Desulfovibrio*, (3) *Mucispirillum*, and (4) *Eubacterium xylanophilum*.

Further random forest models were used to rank features with significant contributions toward group classification, which revealed that at the family level, the mean decrease in accuracy of the Deferribacteraceae, Desulfovibrionaceae, and Clostridiales vadin BB60 group was high (**Supplementary Figure S4**).

Collectively, these findings revealed the dysbiosis in the gut microbiota composition, with specific taxa, linked to CIA mice.

### 3.5 Disparities of microbiome between mice with mild and severe arthritis

We further analyzed differences in the microbiome of CIA mice depending on the severity of arthritis.

**Figure 5:**
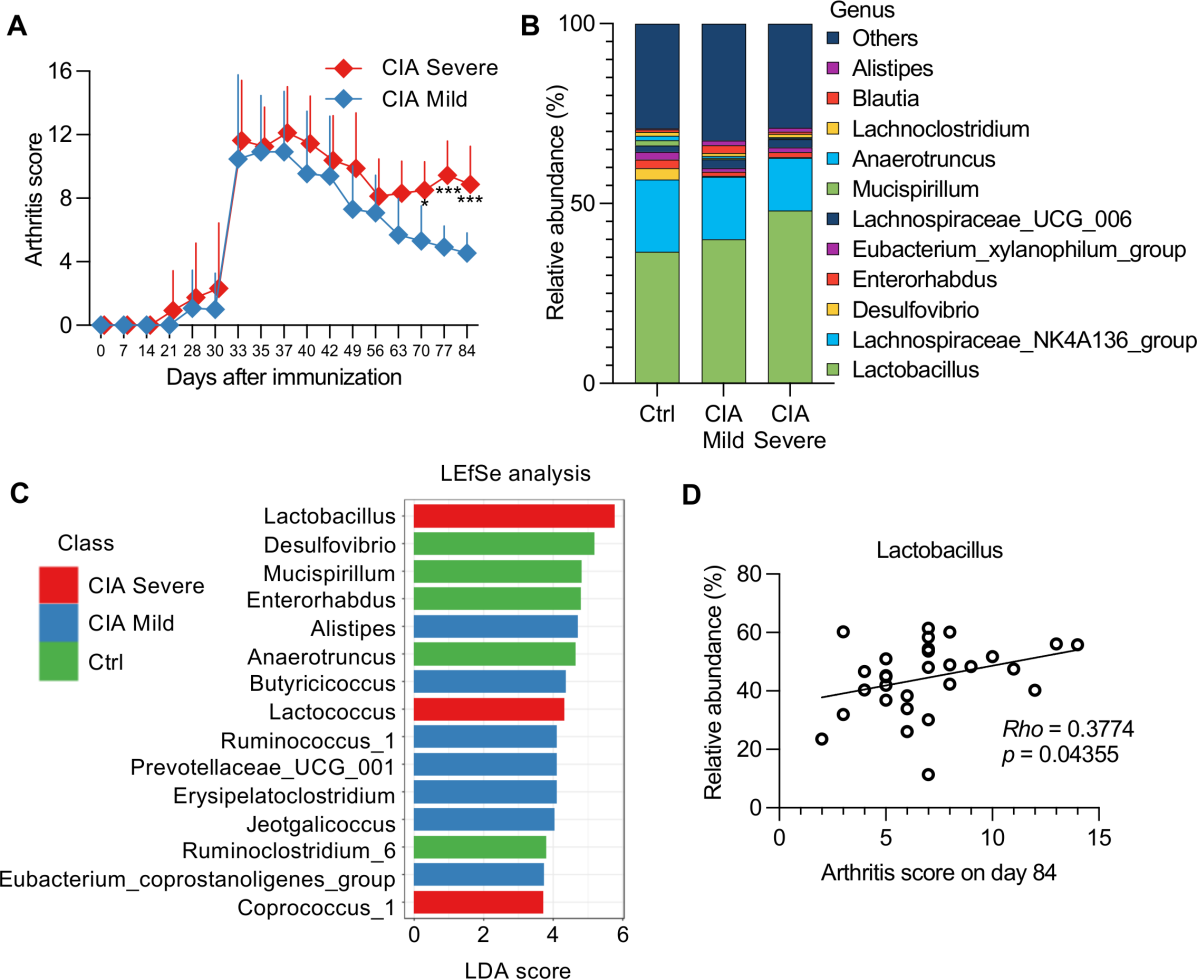
Association between the severity of arthritis and cecal microbiomes in CIA mice. **(A)** CIA mice (*n* = 29) were divided into two groups (severe, *n* = 16; mild, *n* = 13) based on median arthritis scores. Data were analyzed using a two-way repeated-measures ANOVA with Sidak’s multiple-comparison tests. *p < 0.05, ***p < 0.001. **(B)** Taxonomic bar plot showing the altered composition of cecal microbiota in mild or severe CIA mice compared with that in Ctrl mice. Data are shown as the mean relative abundance of bacterial taxa at the genus level (Ctrl group, *n* = 10; mild CIA, *n* = 13; severe CIA, *n* = 16). **(C)** Linear discriminant analysis (LDA) scores from linear discriminant analysis effect size (LEfSe) analysis were assessed at the genus level, depicting the most differentially abundant taxa among the cecal microbiomes of the Ctrl (green), mild CIA (blue), and severe CIA (red) mice. The top 15 taxa with LDA scores >2 and *p*-values <0.05 in the Wilcoxon signed-rank test are shown. **(D)** Correlation between arthritis scores of mice with CIA (*n* = 29) on day 84 and the relative abundance of *Lactobacillus*. Spearman’s correlation coefficient (Rho) and p-values are shown in the graphs.

A total of 16 CIA mice had incomplete remission and experienced a flare-up to high disease activity that exceeded the median value of arthritis scores on day 84 and was defined as severe (**Figure 5A**). In contrast, 13 mice showed a monotonously improved condition, with a mild course (**Figure 5A**; **Supplementary Table S1**).

The Shannon diversity index (microbial richness and evenness) and Simpson indices (mainly considering evenness) in severe CIA mice were lower than those in mild CIA or Ctrl mice (**Supplementary Figure S5A**), which indicated a potential imbalance in the gut microbiome associated with severe disease. Although nonmetric multidimensional scaling expressed dissimilarity in bacterial composition by multidimensional clustering (**Supplementary Figure S5B**), it could not distinguish any specific taxa differentially represented in the mild and severe CIA groups (**Supplementary Table S6**). To further investigate these specific taxa, we analyzed the taxonomic changes and LDA scores using the LEfSe algorithm to identify significant bacteria that were differentially expressed in the mild and severe CIA groups (**Figures 5B, C**).

*Lactobacillus*, *Lactococcus*, and *Coprococcus 1* exhibited enriched abundance in the severe CIA group, which may indicate a disruption in the fermentation balance with lactic acid-producing bacteria associated with arthritis severity. The LDA scores highlighted this result as the principal difference between the CIA groups, whereas the abundance of *Alistipes*, *Butyricicoccus*, *Ruminococcus 1*, *Prevotellaceae UCG-001*, *Erysipelatoclostridium*, *Jeotgalicoccus*, and *Eubacterium coprostanoligenes* increased in the mild CIA group. Additionally, *Anaerotruncus* and *Ruminoclostridium 6*, which adapted to the strict anaerobic environment of the gut, were abundantly added in the Ctrl group.

Moreover, Spearman’s correlation analysis showed a significant correlation between the abundance of *Lactobacillus* and arthritis score on day 84 (**Figure 5D**). However, bivariate correlation analysis can be unreliable owing to the identification of spurious correlations in the form of relative fractions of genes or species. To address this issue, we used the SparCC network application to compute the correlation for the compositional data (**Supplementary Table S7**). The five genera that had a positive correlation with the quartile ranking of arthritis scores (*p*-value <0.05) were as follows: (1) genus *Streptococcus* in family *Streptococcaceae*, (2) genus *Enterorhabdus* in family *Coriobacteriaceae*, (3) genus *Lachnoclostridium* in family *Lachnospiraceae*, (4) genus *Parvibacter* in family *Coriobacteriaceae*, and (5) genus *Lactobacillus* in family *Lactobacillaceae*. However, the following two genera had a negative correlation: (1) genus *Oscillibacter* in family *Ruminococcaceae* and (2) genus *Tyzzerella_3* in family *Lachnospiraceae*. The significant correlations observed between these microbiota abundances and arthritis scores suggested that these taxa may serve as potential biomarkers of disease activity.

### 3.6 Integrating multi-omics analysis between microbiota and microbiota-derived C18 PUFA metabolites

**Figure 6:**
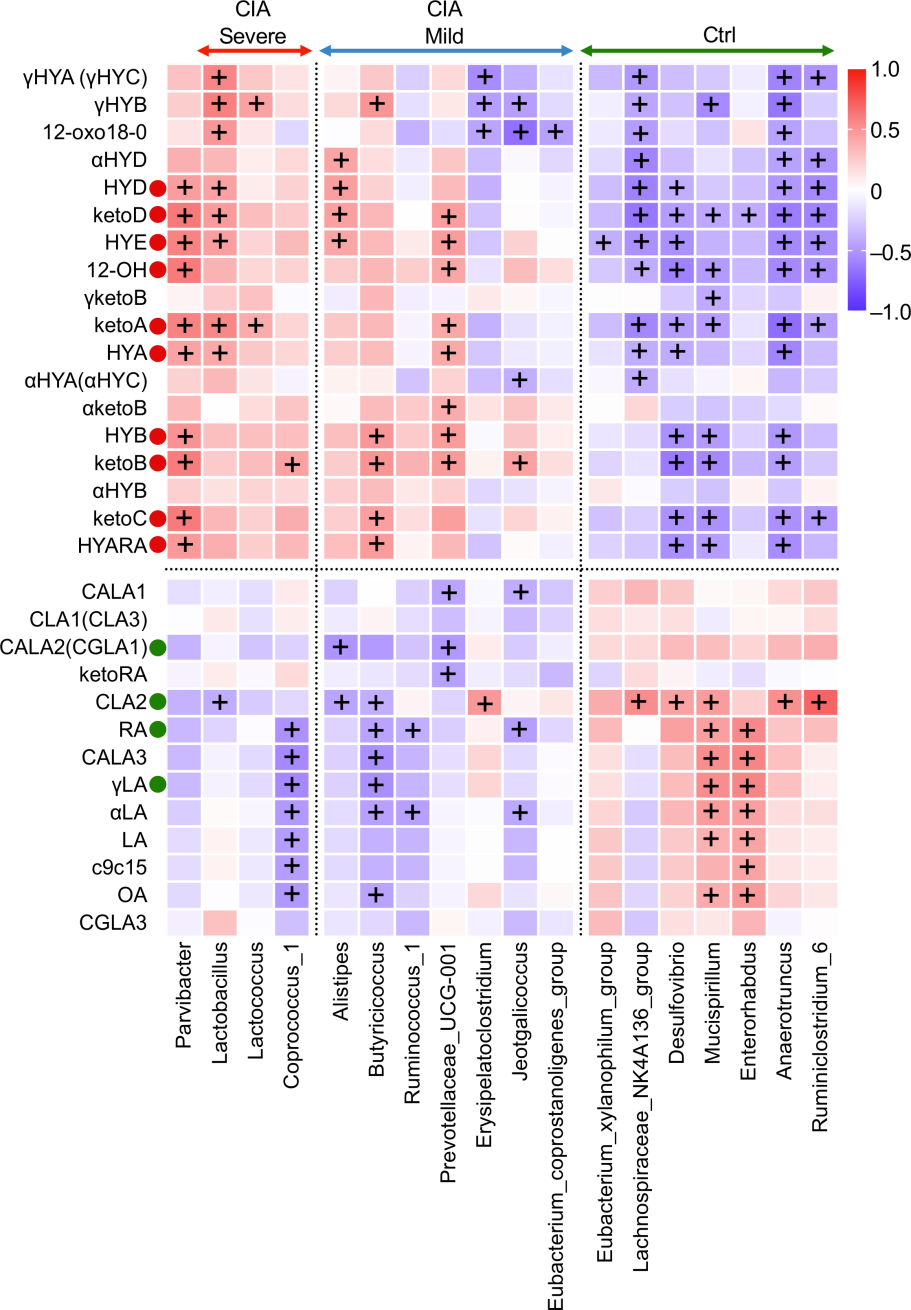
Correlation heatmap elucidating a strong association between cecal microbiota at the genus level and microbiota-derived C18 PUFA metabolites. Spearman’s rank correlation heatmap displays the correlations between cecal microbial families on the x-axis and altered microbiota-derived fatty acid metabolites on the y-axis. The warmer colors represent positive correlations, whereas the cooler colors represent negative correlations. ^+^The adjusted *p*-value (*q*-value) <0.05.

Next, we evaluated the intercorrelations between relative microbial abundance and the concentration of cecal metabolites to identify the bacteria contributing to hydroxyl and oxo production (**Figure 6**; **Supplementary Table S8**).

10, 13 hydroxy and oxo fatty acids (HYA, HYB, HYD, HYE, γHYA, γHYB, γHYC, 12-OH, ketoA, ketoB, ketoC, ketoD, and 12-oxo18-0) displayed positive cross-correlations with *Parvibacter*, *Lactobacillus*, *Lactococcus*, *Alistipes*, and *Prevotellaceae UCG-001* and negative cross-correlations with the *Lachnospiraceae NK4A136* group, *Desulfovibrio*, *Mucispirillum*, *Anaerotruncus*, and *Ruminoclostridium 6*. KetoA, an oxo fatty acid that can identify the separation of the groups and disease activity mentioned in **Figures 2B**, **3**, revealed a strong positive correlation with *Parvibacter*, *Lactobacillus*, *Lactococcus*, and *Prevotellaceae UCG-001*. Interestingly, γHYB showed a positive correlation with *Lactobacillus* and *Lactococcus* and a negative correlation with *Erysipelatoclostridium*, *Jeotgalicoccus*, *Lachnospiraceae NK4A136*, *Mucispirillum*, and *Anaerotruncus*.

CLA2, in contrast to hydroxylated and oxo metabolites, was positively correlated with the abundance of *Lachnospiraceae NK4A136*, *Desulfovibrio*, *Mucispirillum*, *Anaerotruncus*, *Ruminoclostridium 6*, and the family *Clostridiales vadin BB60* group (a family level is not shown in **Figure 6**) and was negatively correlated with the abundance of *Lactobacillus*, *Alistipes*, and *Butyricicoccus.*

LA, OA, γLA, and RA had a significant intercorrelation with the taxa of *Coprococcus 1*, *Butyricicoccus*, *Mucispirillum*, and *Enterorhabdus*. Their absolute values were higher in the Ctrl mice than in the CIA group. However, C18 PUFA substrates had a positive relationship with arthritis scores in the CIA group.

In summary, microbiota-derived intermediate metabolites hydroxylated at positions 10 and 13, which showed increased levels in CIA mice, were positively associated with specific anaerobic genera, such as *Lactobacillus*, *Lactococcus*, and *Prevotellaceae UCG_001*, which showed increased abundance in CIA mice with severe arthritis, and were negatively associated with *Lachnospiraceae NK4A136*, *Desulfovibrio*, *Mucispirillum*, *Anaerotruncus*, and *Ruminoclostridium 6*.

Microbiota-derived C18 PUFA metabolites may be oriented in the presence of enzymes from cecal microorganisms. Tax4fun2 predictive analysis of the functional profiles indicated that the cecum in CIA mice might have a potential source of hydroxylation and decarboxylation processes, such as those performed by oleate hydratase (CLA-HY) and acetoacetate decarboxylase (CLA-DC) (**Supplementary Figure S6**, **Supplementary Table S9**).

## 4 Discussion

In this study, we examined the characteristics of the gut microbiome and C18 PUFA metabolites in CIA mice. To the best of our knowledge, this is the first study to investigate the correlation between microbiota-derived C18 PUFA metabolites and gut dysbiosis integrated with the arthritis severity in a murine CIA model.

We found an association between specific taxonomic differences in the gut microbial communities and the richness of hydroxy or oxo metabolites in the CIA cecum. The hydroxy and oxo metabolites at positions 10 and 13 of LA were associated with the CIA group. Furthermore, γHYB was most significantly associated with arthritis severity and the presence of specific microorganisms, including *Lactobacillus*.

These findings suggest that the chronic accumulation of specific hydroxy or oxo metabolites and *Lactobacillus* in the CIA mice may reflect the ongoing inflammatory processes associated with arthritis. This correlation might suggest a potential role in disease progression and reflect the involvement of oxidative metabolites in gut dysbiosis, highlighting their potential as biomarkers for arthritis severity. However, biological activity on γHYB remains unknown. Further investigation is needed to understand new insights into how the interaction between the gut microbiome and the immune system may influence arthritis.

Several studies on taxonomic differences in CIA mice (28, 29) support the increased abundance of *Lactobacillus* and *Prevotellaceae*, which is consistent with the results of earlier investigations on the abundance of *Lactobacillus*, *Streptococcus*, and *Prevotellaceae* in individuals with rheumatoid arthritis (30–33). The correlation between *Lactobacillus* and hydroxy and oxo metabolites has been demonstrated in gnotobiotic mouse models (17). In the Tax4fun2 functional analysis, CLA-HY and CLA-DC levels were elevated in CIA mice, which suggests that *Lactobacillus* exhibits saturation metabolism on Δ9 and Δ12 double bonds in PUFAs, producing hydroxy and oxo fatty acids at 10 and 13 positions (34).

Regarding plasma metabolites, several host factors, such as malabsorption and consumption, may alter the metabolite levels to reflect dysbiosis or disease activity—for example, the intestine-type fatty-acid-binding protein family tends to be related to *Lactobacillus* abundance in patients with rheumatoid arthritis (35). Systemic inflammation may alter LA metabolism in the host, and the plasma LA concentration is inversely correlated with disease severity (36). Plasma metabolites did not completely reflect changes in cecal metabolites, but they could still be used to estimate cecal metabolites to some extent. Above all, lipophilic metabolites that originate from the gut microbiota possess Treg-inducing activity based on extraction from feces (37), which suggests their potential to influence systemic inflammation. Thus, this study focused on the alterations in cecal metabolites. The decrease in CLA2 levels was similar to that reported in a previous study in patients with rheumatoid arthritis (5), and the cecal abundance of CLA2 tended to decrease with arthritis severity, indicating that CLA2 may be a potential biomarker.

This inverse relationship suggests that higher CLA2 levels may be associated with reduced inflammation or disease severity, potentially pointing to a protective effect of CLA2 against the development or progression of arthritis. Several studies have shown that CLAs can modulate immune responses and suppress inflammatory pathways, potentially contributing to the observed negative correlation. The predominant isomers of CLAs (CLA1 and CLA3) found in dairy products and ruminant meat have various beneficial effects; however, CLA2 also has isomer specificity of CLAs as a bioactive mediator (38), such as an interleukin 1 receptor antagonist, suppressing macrophage adhesion activity and suggesting the potential to regulate inflammation. CLA2 binds to the liver X receptor (LXR) alpha as a dietary agonist (39). LXR facilitates an anti-inflammatory role by inhibiting the differentiation of Th17 cells, which may have regulated arthritis in CIA mice intrinsically (40). However, there are no reports of supplementation with CLA2 alone, and further studies are needed to understand the mechanisms underlying this negative correlation and to investigate whether CLA2 supplementation could modulate arthritis outcomes in clinical settings.

Additionally, when we assessed arthritis-induced foot paw swelling and edema, paw thickness negatively correlated with CLA1 and CLA3 levels in the cecum. Several studies have suggested that a mixture of two CLA isomers (CLA1 and CLA3) may have potential benefits for rheumatoid arthritis by modulating bone formation (13), reducing inflammation (41), and suppressing pain (42). However, no clinical trials of supplementation with CLA1 and CLA3 have ameliorated the severity of arthritis, and administration experiments in a CIA model adopted doses of CLAs that were significantly high for ingestion in humans. Because the therapeutic effects of CLA in CIA mice are inconsistent (43, 44), the interpretation of these results is controversial.

Recently, gut-microbiota-derived PUFA isomerization and intestinal CLAs (CLA1 and CLA3) are essential for CD4+CD8αα+ intraepithelial lymphocytes (IELs) (18), which can contribute to controlling gut inflammation by tissue adaptation of regulatory and intraepithelial CD4+CD8αα+ IELs (45). Although our study did not show evidence of IELs in CIA mice, the lower abundance of intestinal CLAs (CLA1 and CLA3) may influence mucosal immunity and intestinal barrier integrity, contributing to a greater exacerbation of arthritis (46).

From the insights into the metabolic reaction (47–50), the conversion of LA to CLAs can be influenced by the strain of lactic acid bacteria, reaction time, medium, pH value, oxidoreduction cofactors, and concentrations of free LA, resulting in varied ratios of CLA isomers. Therefore, it may be necessary to maintain intestinal health for optimal CLA production, which may underscore the importance of nutrition balance in managing rheumatoid arthritis. However, further investigations are required to confirm the factors influencing CLA isomerization and their potential therapeutic implications.

Thus, we hypothesized that the intermediate hydroxy and oxo products accumulated with the richness of *Lactobacillus* in CIA mice, whereas a few conjugated fatty acids, especially CLA2, showed considerably decreased levels. Cecal metabolites may reflect direct interactions between diet and the gut microbiome.

There are several limitations in this study. First, although we identified specific gut-microbiome-derived C18 PUFA metabolites associated with CIA mice, our study design did not repeat the measurement of biomarkers over disease progression. This lack of longitudinal data limits our ability to fully capture the dynamics of microbiome changes and metabolite fluctuations over time. Second, while our study focused on the CIA model, concerning the ratio of genus *Lactobacillus* in the lactic-acid bacteria, rodents and humans do not have a similar distribution of gut microbiota. Therefore, the relevance of these findings to human disease remains to be validated. Further research using humanized gnotobiotic mouse models will be necessary to determine the translational potential of our findings. Third, in our protocol, we could not quantify the histopathological score due to collecting bilateral hind paws for analyzing the metabolites of gut microbes in the joints. Finally, no robust correspondence was found between the plasma and cecal metabolites in this study. Generally, gut-microbiota-derived metabolites can be further metabolized by host metabolism, which may significantly affect the accumulated metabolites. Although the biomarker gap between Ctrl and CIA mice may be significant, we should not focus solely on microbial-based biomarkers. Future studies should integrate more detailed profiling of host metabolic pathways, gut microbiota, and other external factors that influence biomarkers, such as autoantibodies and inflammatory cytokines.

In conclusion, microbiome-derived C18 PUFA metabolites in CIA mice were different from those in healthy controls, which may correspond to alterations in the gut microbiome with dysbiosis. These results suggest that the gut microbiota and C18 PUFA metabolisms are potential biomarkers for arthritis severity and may provide insights into novel nutrition therapeutic strategies in patients with rheumatoid arthritis.

## Data Availability

The original contributions presented in the study are included in the article/**Supplementary Material**. Further inquiries can be directed to the corresponding author.

## Glossary

**Table:**

**Abbreviation**	**Rational formula**	**IUPAC name**
LA: linoleic acid	c9, c12-18:2	cis-9-cis-12-octadecadienoic acid
HYA	10(OH)c12-18:1	10-hydroxy-cis-12-octadecenoic acid
HYC	10(OH)t11-18:1	10-hydroxy-trans-11-octadecenoic acid
HYB	10(OH)-18:0	10-hydroxy-octadecanoic acid
ketoA	10(oxo)c12-18:1	10-oxo-cis-12-octadecenoic acid
ketoC	10(oxo)t11-18:1	10-oxo-trans-11-octadecenoic acid
ketoB	10(oxo)-18:0	10-oxo-octadecanoic acid
CLA1	c9, t11-18:2	cis-9-trans-11-octadecadienoic acid
CLA2	t9, t11-18:2	trans-9-trans-11-octadecadienoic acid
CLA3	t10, c12-18:2	trans-10-cis-12-octadecadienoic acid
10t	t10-18:1	trans-10-octadecenoic acid
OA: oleic acid	c9-18:1	cis-9-octadecenoic acid
13(OH)LA (HYD)	13(OH)c9-18:1	13-hydroxy-cis-9-octadecenoic acid
13(oxo)LA (ketoD)	13(oxo)c9-18:1	13-oxo-cis-9-octadecenoic acid
10,13(diOH)LA (HYE)	10,13(diOH)-18:0	10,13-dihydroxy-octadecanoic acid
αLA: α-linoleic acid	all cis-9,12,15-18:3	all-cis-9,12,15-octadecatrienoic acid
αHYA	10(OH)c12, c15-18:2	10-hydroxy-cis-12-cis-15-octadecadienoic acid
αHYC	10(OH)t11, c15-18:2	10-hydroxy-trans-11-cis-15-octadecadienoic acid
αHYB	10(OH)c15-18:1	10-hydroxy-cis-15-octadecenoic acid
αketoA	10(oxo)c12, c15-18:2	10-oxo-cis-12-cis-15-octadecadienoic acid
αketoC	10(oxo)t11, c15-18:2	10-oxo-trans-11-cis-15-octadecadienoic acid
αketoB	10(oxo)c15-18:1	10-oxo-cis-15-octadecenoic acid
CALA1	c9, t11, c15-18:3	cis-9-trans-11-cis-15-octadecatrienoic acid
CALA2	t9, t11, c15-18:3	trans-9-trans-11-cis-15-octadecatrienoic acid
CALA3	t10, c12, c15-18:3	trans-10-cis-12-cis-15-octadecatrienoic acid
13(OH)ALA (αHYD)	13(OH)c9, c15-18:2	13-hydroxy-cis-9-cis-15-octadecadienoic acid
**Abbreviation**	**Rational formula**	**IUPAC name**
13(oxo)ALA (αketoD)	13(oxo)c9, c15-18:2	13-oxo-cis-9-cis-15-octadecadienoic acid
10,13(diOH)ALA (αHYE)	10,13(diOH)-18:1	10,13-dihydroxy-cis-15-octadecenoic acid
c9c15	c9, c15-18:2	cis-9, cis-15-octadecadienoic acid
t10c15	t10, c15-18:2	trans-10, cis-15-octadecadienoic acid
γLA: γ-linoleic acid	all cis-6,9,12-18:3	cis-6-cis-9-cis-12-octadecatrienoic acid
γHYA	10(OH)c6, c12-18:2	10-hydroxy-cis-6-cis-12-octadecadienoic acid
γHYB	10(OH)c6-18:1	10-hydroxy-cis-6-octadecenoic acid
γHYC	10(OH)c6, t11-18:2	10-hydroxy-cis-6-trans-11-octadecadienoic acid
γketoA	10(oxo)c6, c12-18:2	10-oxo-cis-6-cis-12-octadecadienoic acid
γketoB	10(oxo)c6-18:1	10-oxo-cis-6-octadecenoic acid
γketoC	10(oxo)c6, t11-18:2	10-oxo-cis-6-trans-11-octadecadienoic acid
CGLA1	c6, c9, t12-18:3	cis-6, cis-9, trans-11-octadecatrienoic acid
CGLA2	c6, t9, t12-18:3	cis-6, trans-9, trans-11-octadecatrienoic acid
CGLA3	c6, t9, c12-18:3	cis-6, trans-10, cis-12-octadecatrienoic acid
13(OH)GLA (γHYD)	13(OH)c6, c9-18:2	13-hydroxy-cis-6-cis-9-octadecadienoic acid
13(oxo)GLA (γKetoD)	13(oxo)c6, c9-18:2	13-oxo-cis-6-cis-9-octadecadienoic acid
10,13(diOH)GLA (γHYE)	10,13(diOH)-c6-18:1	10,13-dihydroxy-cis-6-octadecenoic acid
c6c9	c6, c9-18:2	cis-6, cis-9-octadecadienoic acid
c6t10	c6, t10-18:2	cis-6, trans-10-octadecadienoic acid
RA: ricinoleic acid	12(OH)c9-18:1	(9Z,12R)-12-hydroxy-cis-9-octadecenoic acid
HYARA	10, 12(diOH)-c9-18:1	10, 12-dihydroxy-cis-9-octadecenoic acid
ketoRA	12(oxo)c9-18:1	12-oxo-cis-9-octadecenoic acid
12-OH	12(OH)-18:0	12-hydroxyoctadecanoic acid
12-oxo18-0	12(oxo)-18:0	12-oxo-octadecanoic acid
