# Supplementary material for: Impact of dysregulated microbiota-derived C18 polyunsaturated fatty acid metabolites on arthritis severity in mice with collagen-induced arthritis
Source: Front Immunol. 2025 Jan 9;15:1444892. doi: 10.3389/fimmu.2024.1444892 (PMC11754244; doi:10.3389/fimmu.2024.1444892)
Supplement: Supplementary file 1 [file DataSheet1.docx]

Supplementary Material

**Impact of Dysregulated Microbiota-derived C18 Polyunsaturated Fatty Acid Metabolites on Arthritis Severity in Mice with Collagen-induced Arthritis**

**Katsuhiko Yoneda, Sho Sendo, Takaichi Okano, Hidenori Shimizu, Hirotaka Yamada, Keisuke Nishimura, Yo Ueda, Jun Saegusa***

*** Correspondence:** Jun Saegusa: jsaegusa@med.kobe-u.ac.jp

**1 Supplementary Materials**

# Supplementary Figures and Tables

Supplementary tables (.xlsx) are uploaded separately on submission

## Supplementary Figures legends

**Supplementary Figure 1. Clinical course of CIA mice.**

DBA/1J mice were immunized with bovine type II collagen emulsified with complete Freund’s adjuvant on day 0. The CIA mice were injected with lipopolysaccharide (LPS) to synchronize their arthritis onset after the 28th day.

**(A)** Changes in the average body weight in the CIA and Ctrl mice.

**(B)** Changes in the average four-paw thickness in the CIA and Ctrl mice after day 14.

**(C)** Incidence of arthritis in the CIA and Ctrl mice.

The data present the means ± SD. The data in a and b were analyzed using two-way repeated-measures ANOVA with Sidak’s multiple comparisons tests. **p < 0.01, ***p < 0.001, ****p < 0.0001

**Supplementary Figure 2. Alteration of other microbiota-derived C18 PUFA metabolites in the cecum**

**(A)** The scatterplot with jitter indicates the values of cecal metabolites quantified using LC/MS/MS in the Ctrl (green dots) and CIA (red dots) groups. The directing dashed or left–right arrows mean a one-way or two-way linkage of metabolites representing microbiota-derived metabolites from C18 dietary PUFAs. The HY or hydroxyl form (-OH) prefix means hydroxy fatty acids (HYA, HYB, HYC, 12-OH, 13-OHLA, 10,13-OHLA, and HYARA). Similarly, the prefix of Keto or carbonyl form (-oxo) means oxo fatty acids (ketoA, ketoB, ketoC, and 13-oxoLA). The C prefix means conjugated fatty acids (CLA1, CLA2, CLA3). Data could not be obtained for a few metabolites under the measurement limit, resulting in no data.

**(B)** The scatterplot with jitter indicates the values of cecal metabolites from α-linoleic acid quantified using LC/MS/MS in the Ctrl (green dots) and CIA (red dots) groups.

**(C)** The scatterplot with jitter indicates the values of cecal metabolites from γ-linoleic acid quantified using LC/MS/MS in the Ctrl (green dots) and CIA (red dots) groups.

**(D)** The scatterplot with jitter indicates the values of cecal metabolites from ricinoleic acid and 12-hydroxy octadecanoic acids quantified using LC/MS/MS in the Ctrl (green dots) and CIA (red dots) groups.

The data are described as the medians with interquartile ranges and were analyzed using the Mann–Whitney U test. **p < 0.01, ***p < 0.001, ****p < 0.0001

**Supplementary Figure 3. CLA2 in plasma and cecum tended to be negatively correlated with arthritis severity.**

Scatterplots present the relationships between arthritis scores on the x-axis and CLA2 levels (a) in the plasma of CIA mice (n=5) and (b) the cecum of CIA mice (n=29) on the y-axis. Each straight line means the regression line that best fits the points in the scatterplot, which were analyzed using Pearson’s product-moment correlation coefficient.

**Supplementary Figure 4. Random forest model of the cecal microbiomes between CIA and Ctrl mice at the family level.**

A predictive model was calculated using cecal microbiomes altered (p < 0.05) between CIA and Ctrl mice. Taxa are ranked based on their importance for group classification based on the mean decrease accuracy, displayed on the y-axis. Red and blue boxes indicate high or low abundance in the CIA and Ctrl groups, respectively.

**Supplementary Figure 5. Gut dysbiosis is pronounced in CIA mice with severe arthritis.**

CIA mice were divided into two groups based on arthritis scores: severe (n=16) and mild (n=13).

**(A)** Alpha diversity indices (Shannon and Simpson index) among groups of Ctrl, mild CIA, and severe CIA mice. Data are presented as medians with interquartile ranges and were analyzed using the Kruskal–Wallis test and multiple comparison tests by controlling the false discovery rate (FDR) using the Benjamini and Hochberg method. *p < 0.05, **p < 0.01

**(B)** Nonmetric multidimensional scaling (NMDS) plots are presented for beta diversity using Bray–Curtis index (left) and Jensen–Shannon divergence (right). The dots are colored by control (Ctrl), CIA mild, and CIA severe mice. Stress values are displayed in the figure. ANOSIM and PERMANOVA analyses are available in Supplementary Table 5.

**Supplementary Figure 6. Prediction of functional profiles and functional redundancy based on 16S rRNA gene sequences.**

Functional predictive analysis by Tax4Fun2 suggests the potential function of bacteria associated with fatty acid metabolism. Scatterplots with jitter show the predictive gene abundance values for Ctrl (green dots) and CIA (red dots) mice. There are three types of enzyme, fatty acid hydratase (CLA-HY, EC 4.2.1.53), hydroxy fatty acid dehydrogenase (CLA-DH, EC 1.1.1.98), and acetoacetate decarboxylase (CLA-DC, EC 4.1.1.4), that are involved in a multi-step reaction consisting of hydration, dehydrogenation, double-bond shift, and dehydration. Reaction scheme of linoleic acid isomerization to CLA. Oleate hydratase (CLA-HY) converts linoleic acid (LA) to 10-hydroxy-cis-12-octadecenoic acid (HYA). Acetoacetate decarboxylase (CLA-DC) converts 10-oxo-12(Z)-octadecenoic acid (KetoA) to 10-oxo-11(E)-octadecenoic acid (KetoC). Hydroxy fatty acid dehydrogenase (CLA-DH) could not be predicted. Data were analyzed using the Mann–Whitney U test and are described as medians with interquartile ranges. *p < 0.05.
