# Supplementary figures and images for: Impact of dysregulated microbiota-derived C18 polyunsaturated fatty acid metabolites on arthritis severity in mice with collagen-induced arthritis
Source: Front Immunol. 2025 Jan 9;15:1444892. doi: 10.3389/fimmu.2024.1444892 (PMC11754244; doi:10.3389/fimmu.2024.1444892)

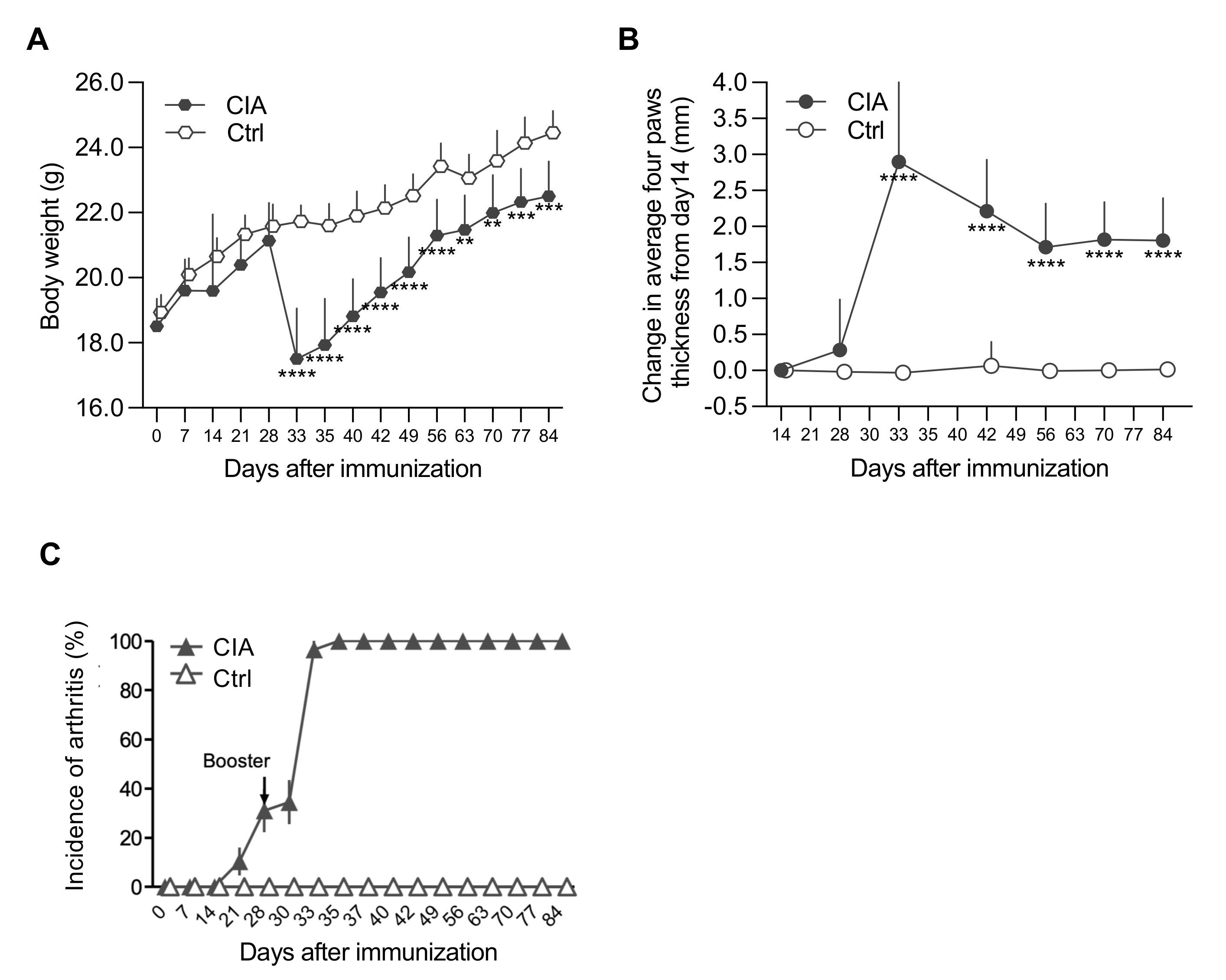

Supplement: Supplementary file 3 [file Image1.tif]

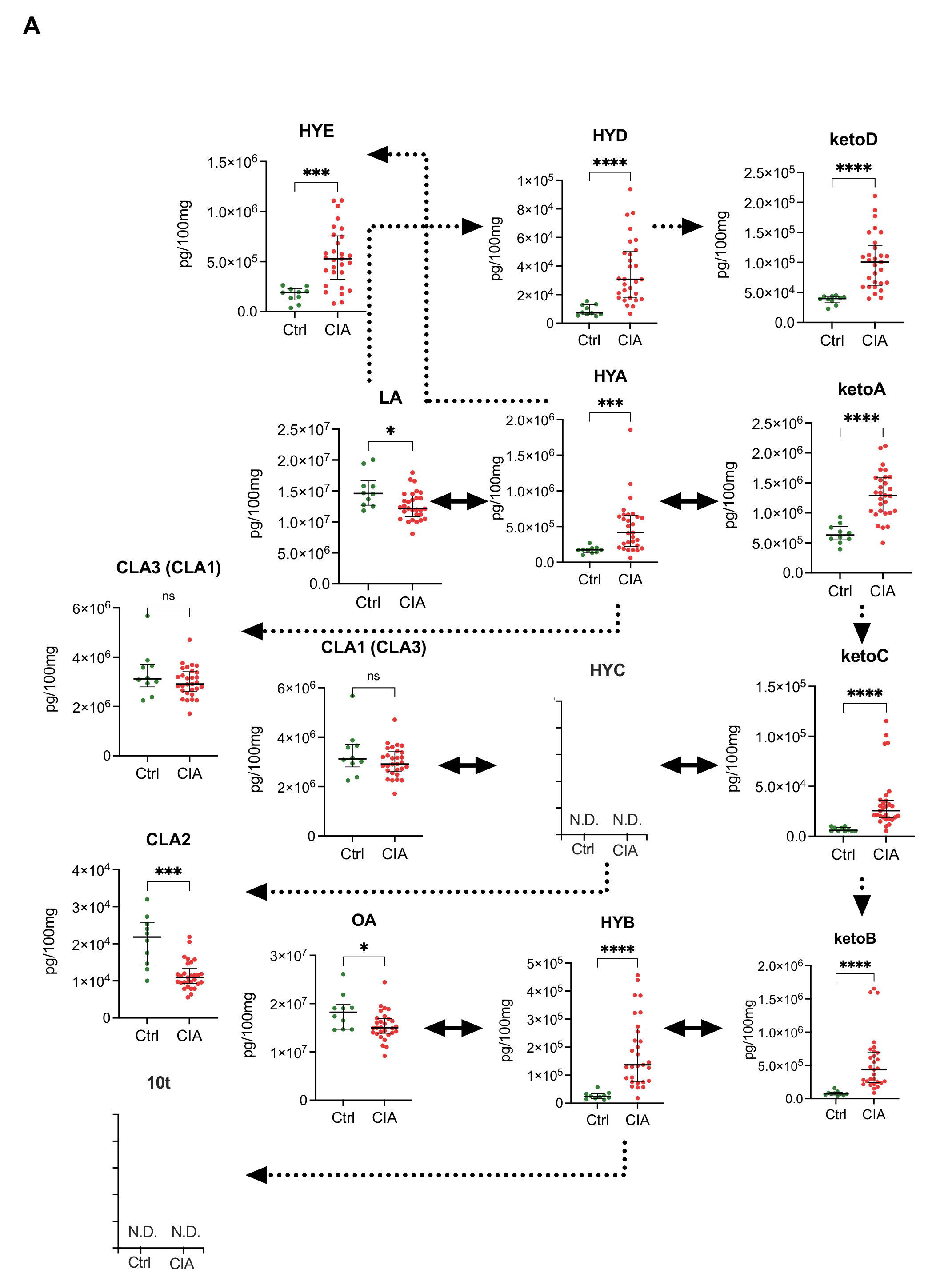

Supplement: Supplementary file 4 [file Image2.tif]

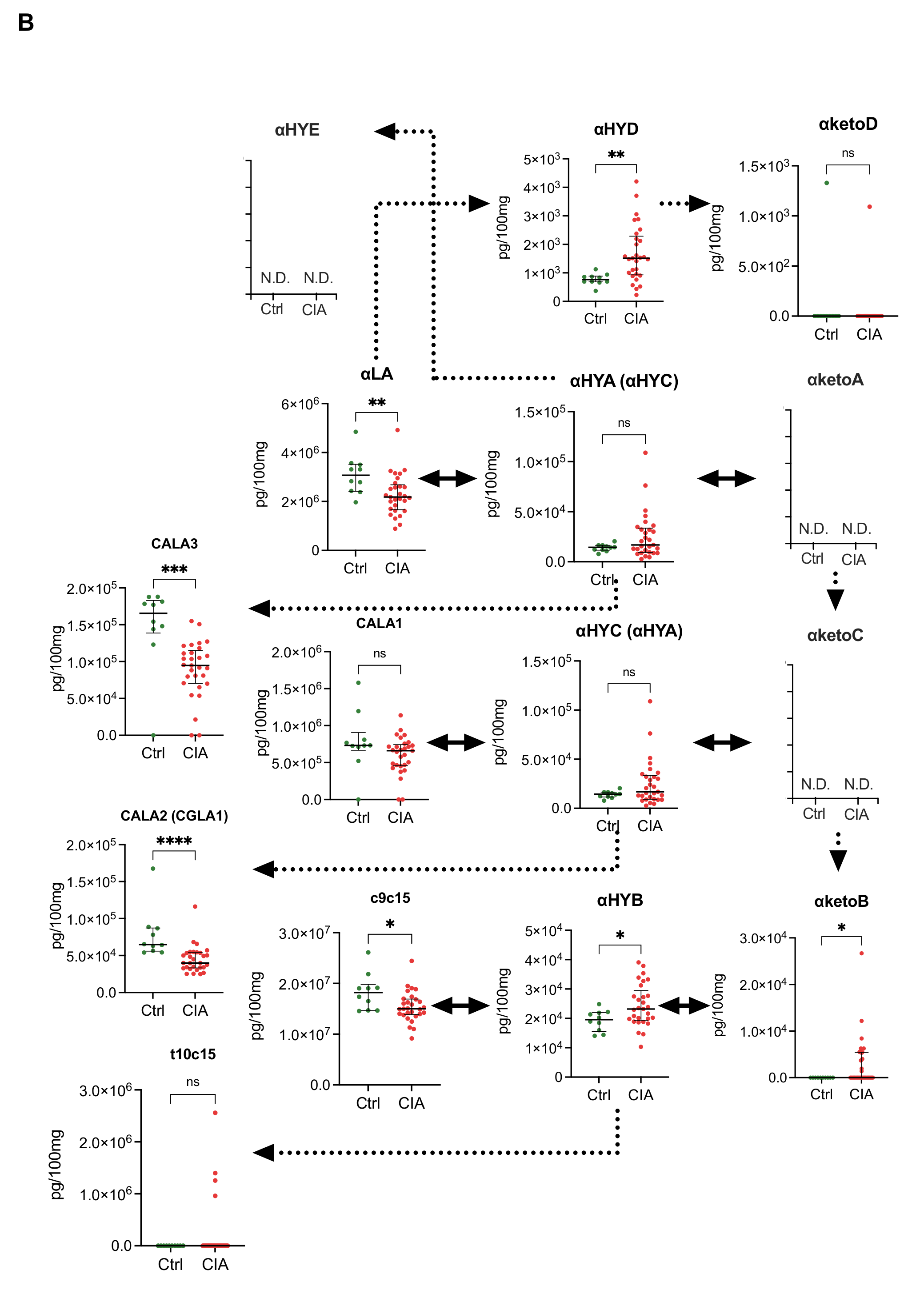

Supplement: Supplementary file 5 [file Image3.tif]

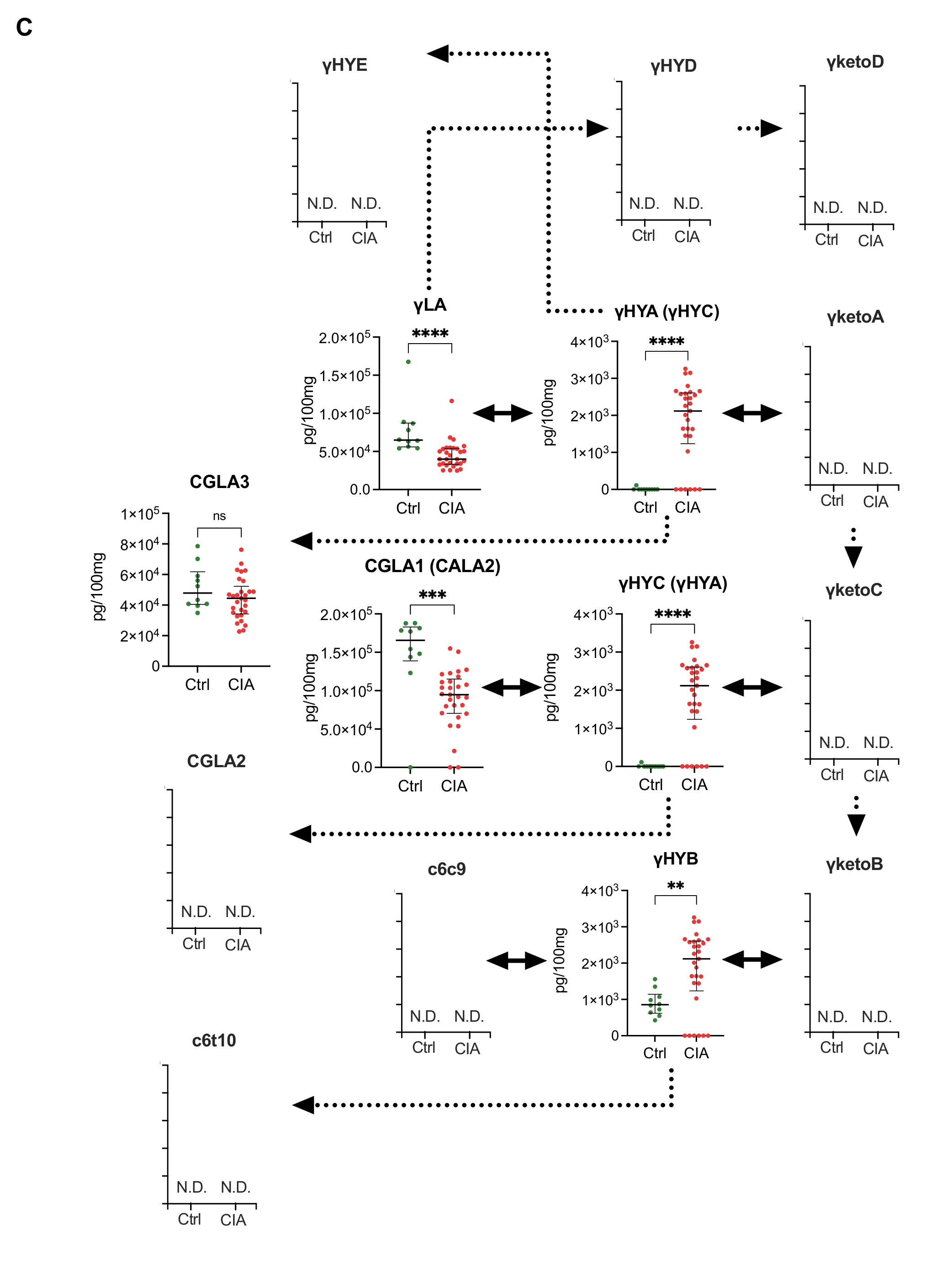

Supplement: Supplementary file 6 [file Image4.tif]

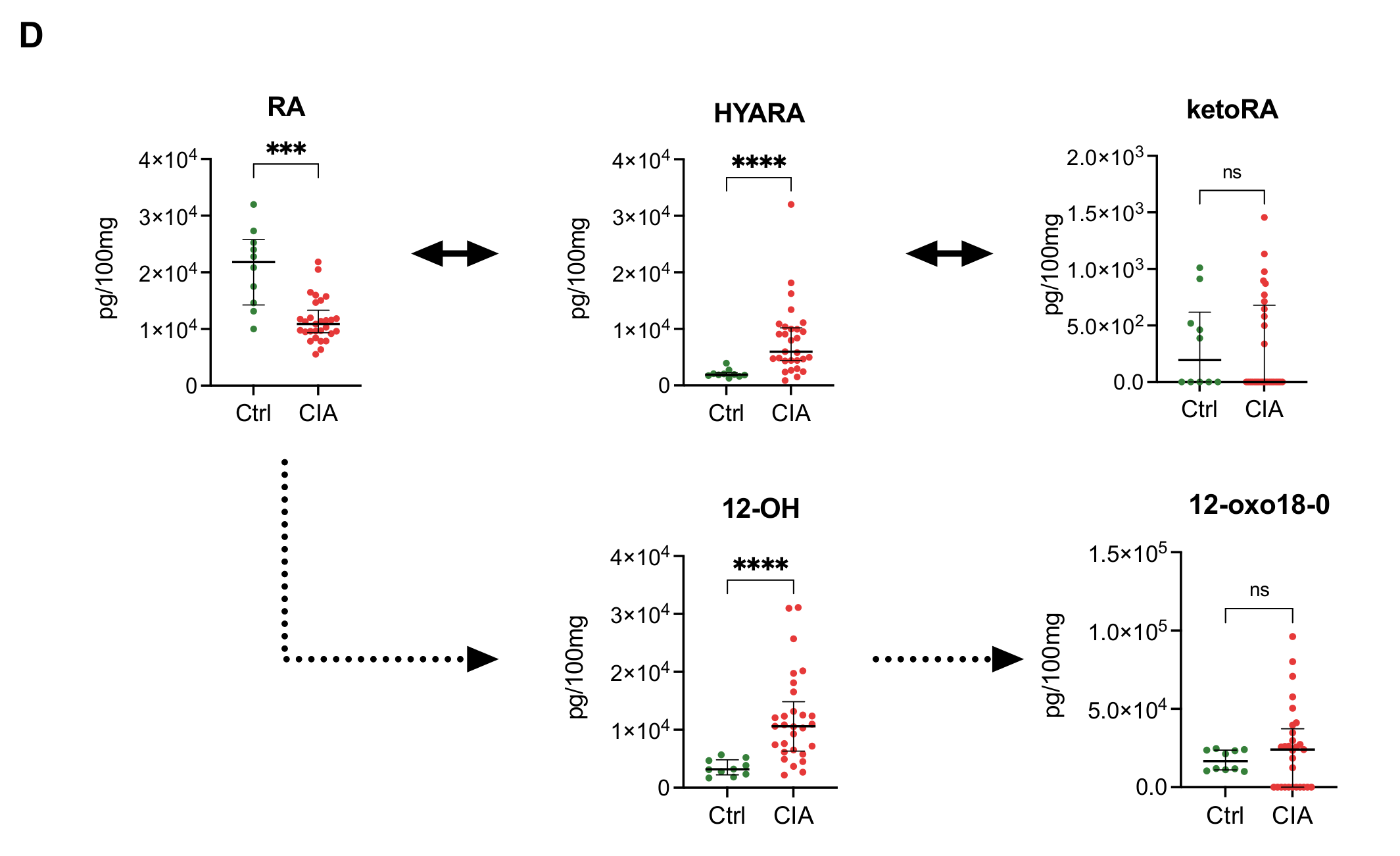

Supplement: Supplementary file 7 [file Image5.tif]

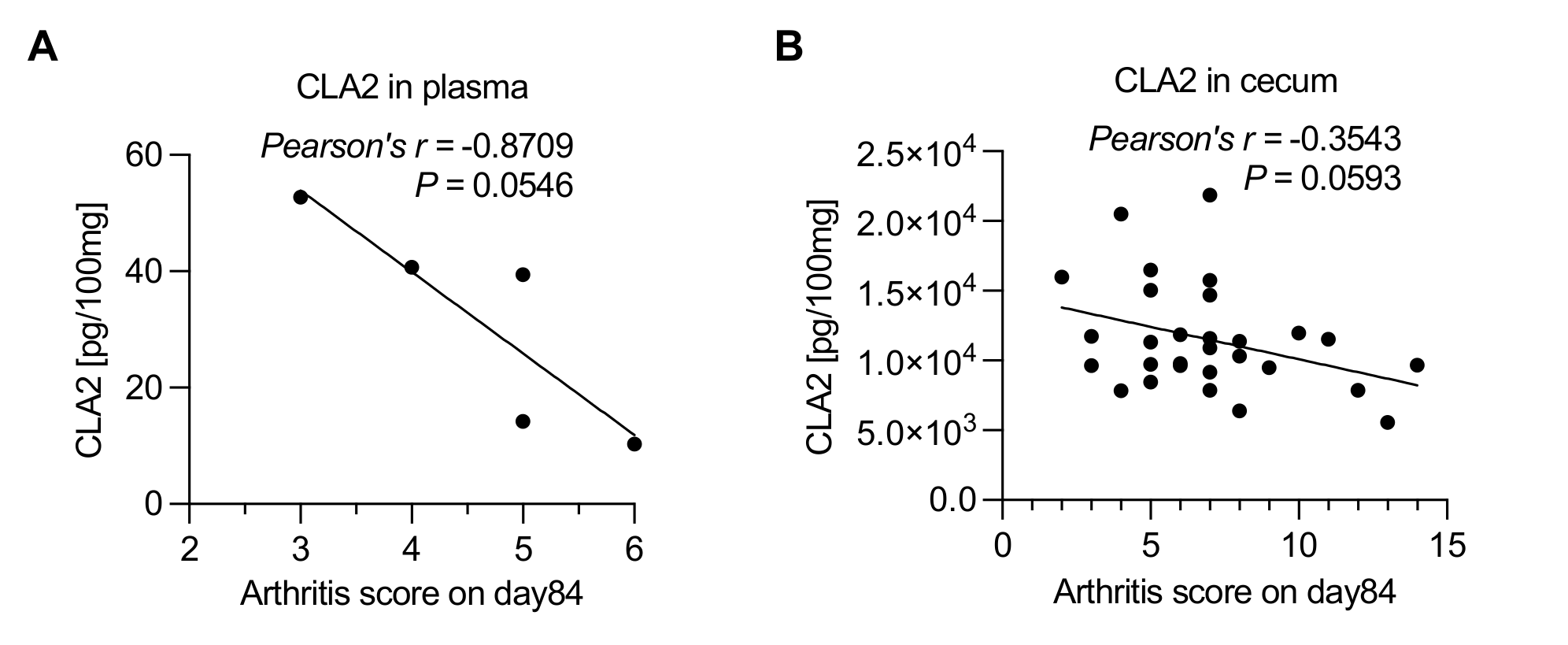

Supplement: Supplementary file 8 [file Image6.tif]

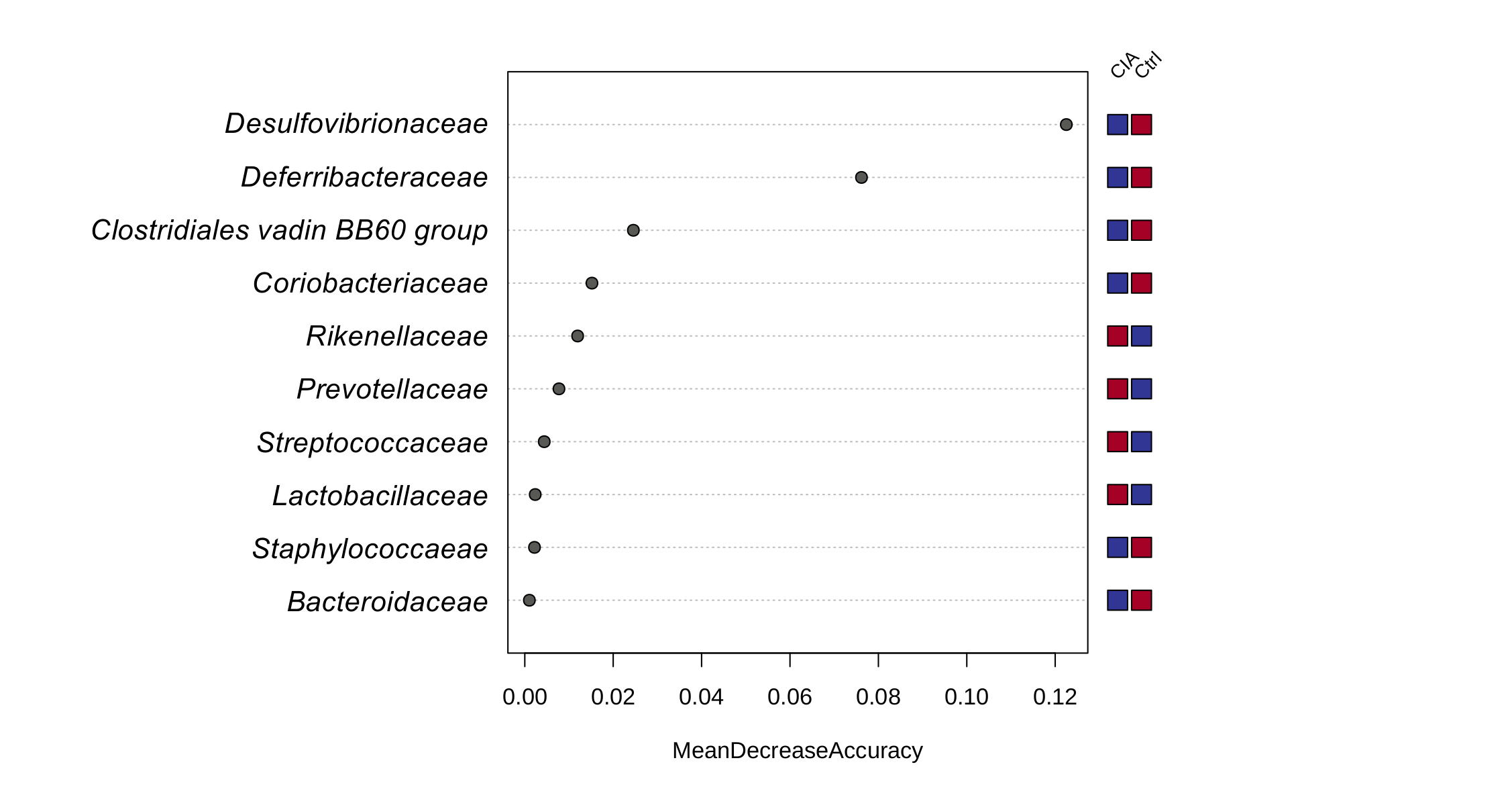

Supplement: Supplementary file 9 [file Image7.tif]

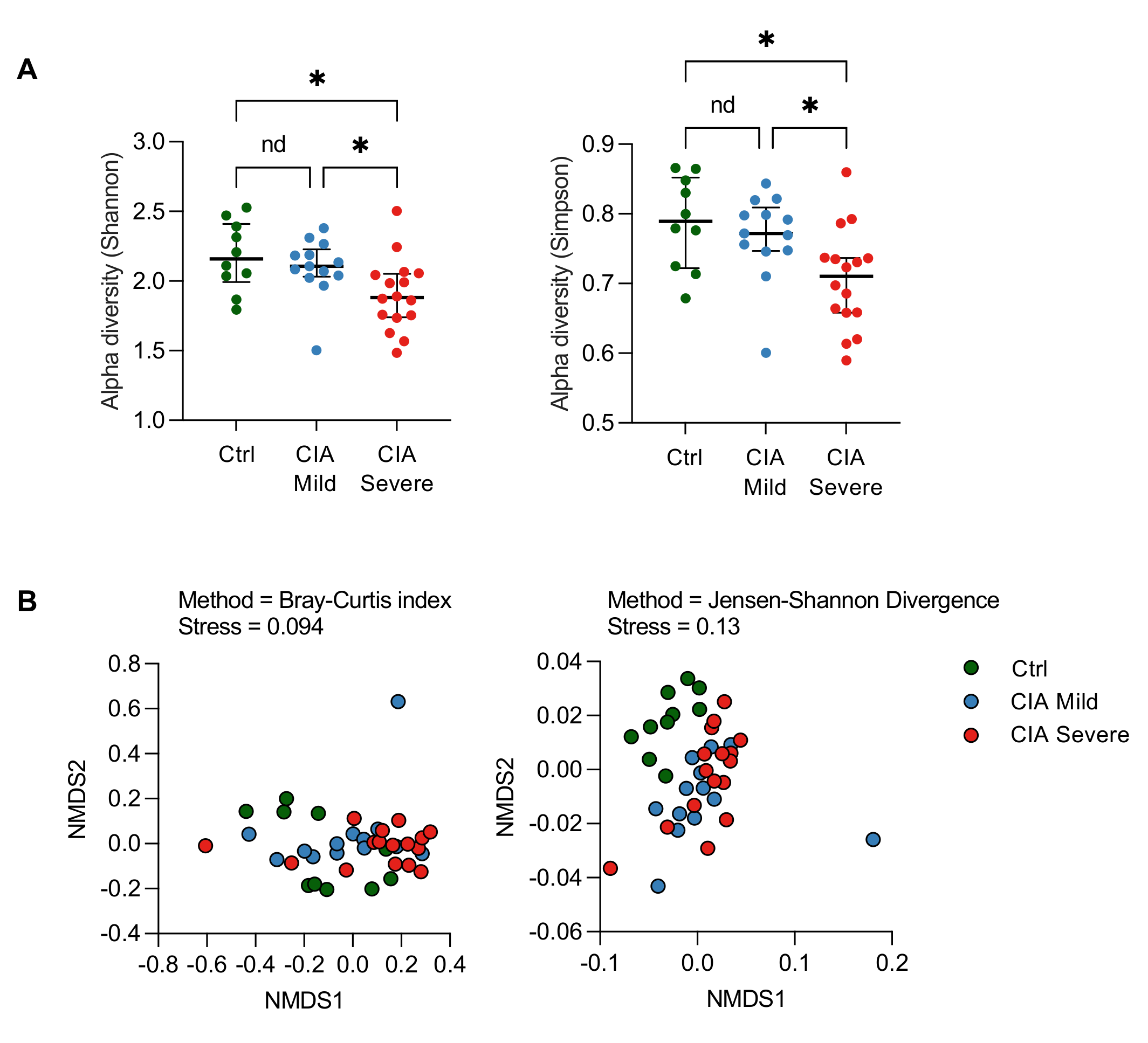

Supplement: Supplementary file 10 [file Image8.tif]

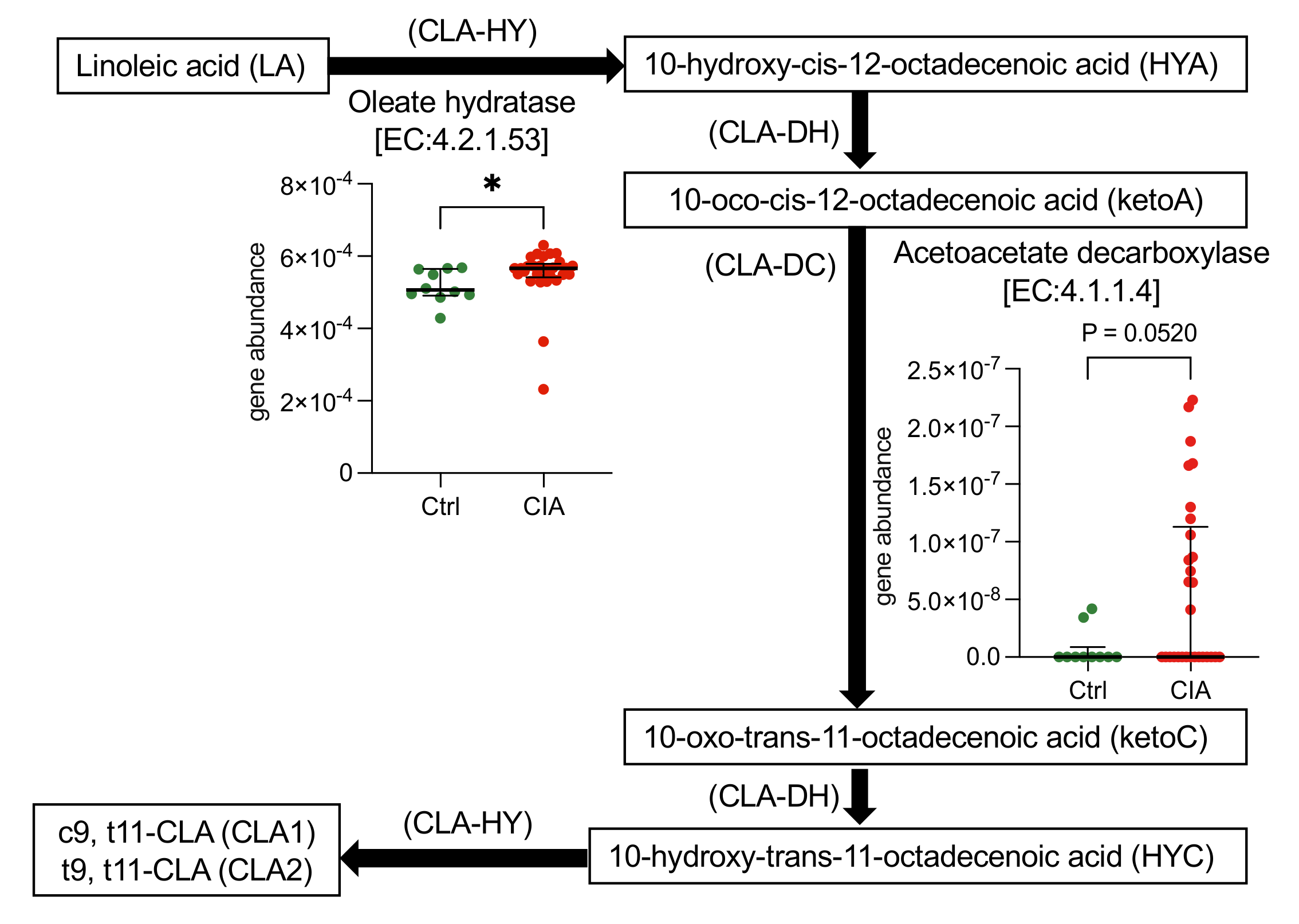

Supplement: Supplementary file 11 [file Image9.tif]
